# The Chemical Constituents of Diaphragma Juglandis Fructus and Their Inhibitory Effect on α-Glucosidase Activity

**DOI:** 10.3390/molecules27103045

**Published:** 2022-05-10

**Authors:** Jinyan Tan, Yangang Cheng, Shihui Wang, Jianli Li, Haiqin Ren, Yuanbiao Qiao, Qingshan Li, Yingli Wang

**Affiliations:** 1 Shanxi Modern Chinese Medicine Engineering Laboratory, Shanxi University of Chinese Medicine, Jinzhong 030619, China; 15636833827@163.com (J.T.); chengyg1992@163.com (Y.C.); wangshihui9516@163.com (S.W.); lijianli1124ljl@163.com (J.L.); haiqinren@163.com (H.R.); 2 Shanxi Key Laboratory of Innovative Drug for the Treatment of Serious Diseases Basing on the Chronic Inflammation, Shanxi University of Chinese Medicine, Jinzhong 030619, China; qiaoyb@sxtcm.edu.cn

**Keywords:** walnut, megastigmanes, bicyclic neomegastigmane, flavonoids, phenylpropanoids, α-glucosidase inhibition activity

## Abstract

In our current investigation, 37 constituents (**1**–**37**), including 11 megastigmanes (**1**–**11**), 17 flavonoids (**12**–**28**) and 9 phenylpropanoids (**29**–**37**), were isolated from a 70%-EtOH extract of Diaphragma juglandis Fructus. Among them, compounds **1**–**3**, **12** and **29** were new compounds and their structures were elucidated on the basis of physicochemical evidence and meticulous spectroscopic analysis (NMR, HRESIMS and CD). Compounds **13**, **16**, **21** and **28** showed moderate inhibitory effect on α-glycosidase inhibitory activities, with IC_50_ values being in the range of 29.47–54.82 µM and stronger than the positive control (acarbose, 60.01 ± 4.82 µM).

## 1. Introduction

The walnut (*Juglans regia* L.) is consumed globally as a high economic value crop [1]. The edible portion of walnuts (kernel) has been processed into many types of foods due to its unique and highly nutritious nature and health-related benefits [2]. However, Diaphragma juglandis Fructus, the dry wooden diaphragm inside the walnut, mainly consists of undigestible fiber and lignin and is usually discarded as waste during the processing of the walnut [3]. In fact, Diaphragma juglandis Fructus is a traditional Chinese medicine and has been used to treat several illnesses such as insomnia, diarrhea, kidney deficiency and reproductive diseases for a long time [4,5]. It has also been used as an herbal tea and a dietary supplement in folk culture [6]. The research shows that Diaphragma juglandis Fructus is rich in a variety of bioactive components, such as flavonoids, saponins, phenolic acids and polysaccharides [7].

**Figure 1:**
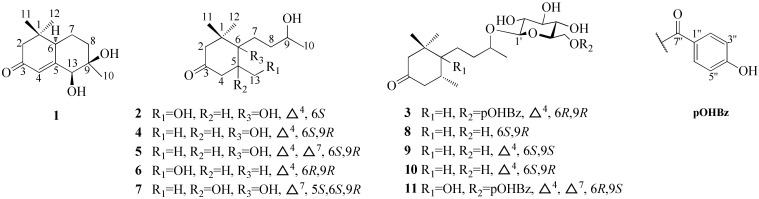
The structures of megastigmanes (**1**–**11**) isolated from Diaphragma juglandis Fructus.

Previously, our team focused on the antidiabetic effect of Diaphragma juglandis Fructus, and it was found to improve symptoms of diabetes via the AKT/FoxO1 signaling pathway [8]. As part of our continuous program to identify new potential candidates to control diabetes using natural products, the subsequent phytochemical study led to the isolation of 37 constituents (**1**–**37**) from Diaphragma juglandis Fructus, including 11 megastigmanes (**1**–**11**) (Figure 1), (**12**–**28**) and **9** phenylpropanoids (**29**–**37**). Among them, compounds **1**–**3**, **12** and **29** were new compounds. Their structures were elucidated on the basis of physicochemical evidence, in-depth NMR spectroscopic analysis and high-resolution mass spectrometry. Meanwhile, the α-glucosidase inhibition activities of all isolates were evaluated. The isolation, structural identification and bioactivity evaluation of the obtained compounds are reported herein.

## 2. Results and Discussion

### 2.1. Structural Elucidation

**Figure 2:**
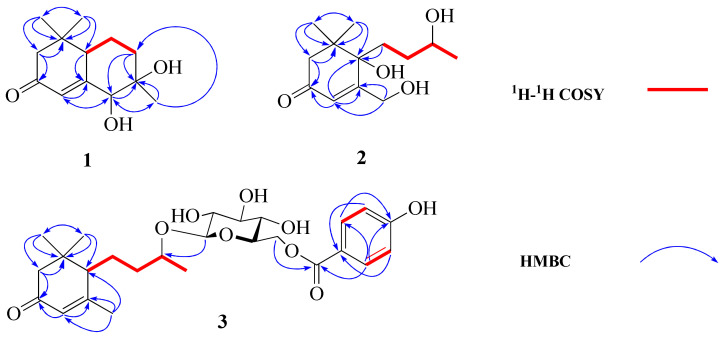
^1^H-^1^H COSY and key HMBC correlations of compounds **1**–**3**.

**Figure 3:**
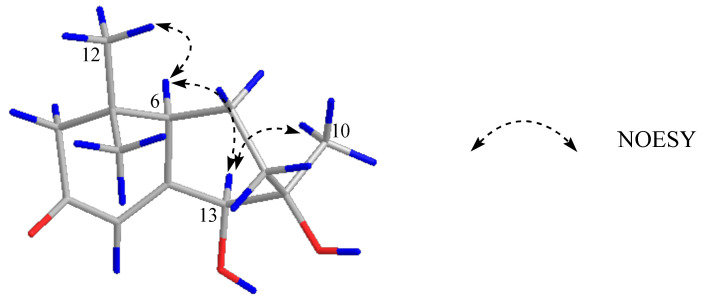
Key NOESY correlations of compound **1**.

**Figure 4:**
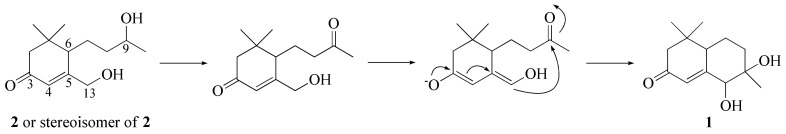
Plausible biogenetic pathway for compound **1**.

Compound **1** displayed a quasi-molecular ion peak at *m/z* 225.1489 ([M+H]^+^, calcd 225.1490), in agreement with the molecular formula C_13_H_20_O_3_, corresponding to four degrees of unsaturation. The analysis of ^1^H-NMR data (Table 1) revealed that **1** possessed three methyls [*δ*_H_ 1.00, 1.03, 1.05 (3H each, both s, H_3_-10, 11, 12)], three methylenes and a methine bearing an oxygen function [*δ*_H_ 4.08 (1H, d, *J* = 1.4 Hz, H-13)]. In addition, the ^13^C-NMR (Figure S4) and DEPT spectra revealed a quaternary carbon bearing an oxygen function (*δ*_C_ 76.8), a trisubstituted olefin [*δ*_H_ 6.15 (1H, br s, H-4), *δ*_C_ 121.0 (C-4), 168.2 (C-5)] and a conjugated carbonyl carbon (*δ*_C_ 202.6). The planar structure of **1** was determined to be a bicyclic neomegastigmane skeleton through the interpretation of various NMR experiments [9], including HSQC, ^1^H-^1^H COSY and HMBC spectra. Namely, the ^1^H-^1^H COSY experiments of **1** indicated the presence of one partial written in red bold lines (Figure 2). In addition, obvious long-range correlations were observed between the following proton and carbon pairs in the HMBC experiments: H_2__-_2 and C-1, 3, 6; H-4 and C-5, 6, 13; H-6 and C-5; H_2_-8 and C-6, 9, 13; H-13 and C-4, 5, 9, 10; H_3_-10 and C-8, 9, 13; H_3_-11 and C-1, 2, 6, 12; H_3_-12; and C-1, 2, 6, 11 (blue arrow in Figure 2). Next, the relative stereostructure of **1** was clarified to be 9*β*-OH and 13*β*-OH by the NOESY experiment, in which correlations were observed between the following proton pairs: H_3_-12 and H-6; H-6 and H-13; H-13; and H_3_-10 (Figure 3). Based on the above-mentioned evidence, the structure of **1** was elucidated to be a bicyclic megastigmane named diamegastigmane A, as shown in Figure 1. Bicyclic megastigmanes are a small but growing group of natural products, and a possible biosynthetic pathway for **1** is further proposed in this paper (Figure 4).

Compound **2** was assigned a molecular formula of C_13_H_22_O_4_ through an analysis of the HRESIMS ion peak at *m/z* 485.3115 ([2M+H]^+^, calcd 485.3114). The ^1^H-NMR (Table 1) spectrum showed the presence of two methyl singlets [*δ*_H_ 1.04, 1.09 (3H each, both s, H_3_-11, 12)], a doublet methyl singlet [*δ*_H_ 1.16 (3H, d, *J* = 6.2 Hz, H_3_-10)], a methine and methylene bearing a hydroxyl group [*δ*_H_ 3.67 (1H, m, H-9), 4.39 (2H, t, *J* = 1.9 Hz, H_2_-13)] and a trisubstituted olefin [*δ*_H_ 6.10 (1H, br. s, H-4)]. The ^13^C-NMR spectrum exhibited 13 carbon signals. In combination with the HSQC spectrum, carbon signals were identified as three methyls [*δ*_C_ 23.5 (C-10), 24.4 (C-11), 23.5 (C-12)], four methylenes [*δ*_C_ 50.8 (C-2), 35.8 (C-7), 34.9 (C-8), 63.1 (C-13)], an oxygenated methine carbon [*δ*_C_ 68.9 (C-9)], a quaternary carbon [*δ*_C_ 43.1 (C-1)], an oxygenated quaternary carbon [*δ*_C_ 78.8 (C-6)], two olefinic carbons [*δ*_C_ 122.4 (C-4), 173.9 (C-5)] and a carbonyl carbon [*δ*_C_ 200.9 (C-3)]. The NMR spectroscopic data (Table 1) for **2** closely resembled those of apocynol B, and further analysis of the 2D NMR spectra revealed that the significant difference was the absence of one olefin in the side chain [10]. The ^1^H-^1^H COSY spectrum of **2** enabled the identification of the H_2_-7/H_2_-8/H-9/H_3_-10 unit. Further, HMBC correlations from H_2_-7 to C-1, C-5 and C-6 arranged the carbon chain that connected to C-6 (Figure 2). Moreover, the CD spectrum indicated a 6*S*-configuration due to a positive Cotton effect at 323 nm and a negative Cotton effect at 272 nm [11]. However, the chirality of C-9 in the side chain was difficult to assign due to the lack of direct evidence and thus, needs to be further determined. Consequently, the structure of **2** was identified (Figure 1) and named diamegastigmane B.

Compound **3** has a molecular formula of C_26_H_36_O_9_ based on the HRESIMS ion at *m/z* 493.2442 ([M+H]^+^, calcd 493.2437). The ^1^H-NMR spectrum (Table 1) of **3** showed two doublet signals at *δ*_H_ 7.89 (2H, d, *J* = 8.8 Hz, H-2″,6″) and 6.83 (2H, d, *J* = 8.8 Hz, H-3″,5″), attributed to the AA′BB′ system in a 1,4-substituted benzene ring, assigned to the 4-hydroxybenzoyl group. The location of the 4-hydroxybenzoyl group was established at C-6′ in the pyranosyl moiety according to the long-range correlation from a proton signal at *δ*_H_ 4.34 (1H, d, *J* = 7.7 Hz, H-1′) to a carbon signal at *δ*_C_ 167.9 (C-7″) in the HMBC spectrum (Figure 2).

The 1D NMR spectroscopic data (Table 1) of 3 showed significant similarity to those of hirtionoside C, except for the replacement of the gallic acid at the 6′-position by a 4-hydroxybenzoic acid [12]. The absolute configuration at the 6-position was confirmed to be *R* by the CD spectrum (positive Cotton effect at 333 nm) [13], and those at the 9-position were also determined to be *R* by comparing ^13^C NMR data according to the *β*-d-glycosylation-induced shift-trend rule [14]. Therefore, the structure of **3** was determined (Figure 1) and named diamegastigmane C.

**Figure 5:**
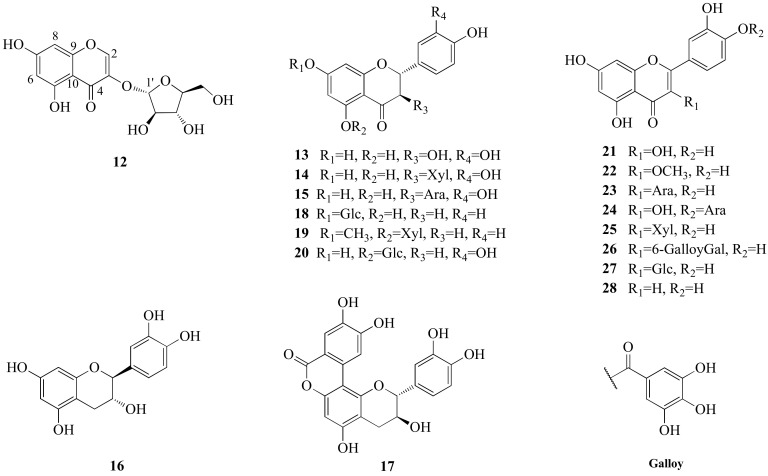
The structures of flavonoids (**12**–**28**) isolated from Diaphragma juglandis Fructus. (Ara = *α*-l-arabinofuranose; Xyl = *β*-d-xylopyranose; Glc = *β*-d-glucopyranose; Gal = *β*-d-galactopyranose).

**Figure 6:**
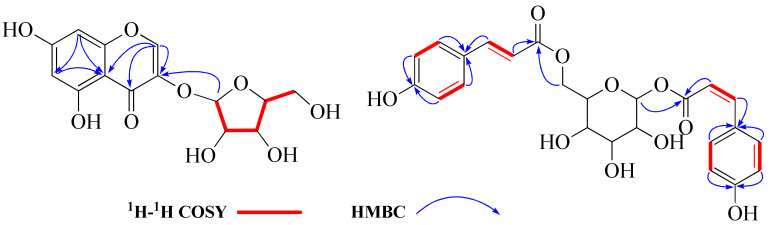
^1^H-^1^H COSY and key HMBC correlations of compounds **12** and **29**.

Compound **12** was obtained as yellow amorphous powder (Figure 5). Its molecular formula was deduced as C_14_H_14_O_9_ on the basis of a quasi-molecular ion peak in HRESIMS (C_14_H_15_O_9_, *m/z* 327.0715 [M+H]^+^, calcd 327.0716). The ^1^H-NMR spectrum of **12** (Table 2) displayed two meta-coupled aromatic protons at *δ*_H_ 6.20 (1H, d, *J* = 2.1 Hz, H-6) and 6.31 (1H, d, *J* = 2.1 Hz, H-8), together with a singlet at *δ*_H_ 8.09 (1H, s, H-2) in the low field. The ^13^C-NMR spectrum (Table 2) showed 14 carbon signals, including 9 aromatic carbon signals and 5 sugar carbon signals. The 1D NMR spectra data of **12** showed a similar pattern to those of 3,5,7-trihydroxylchromone-3-*O*-*α*-l-arabinopyranoside, except for the sugar part [15]. The anomeric carbon of 12 at *δ*_C_ 109.5 (C-1′) and other four sugar carbon signals at *δ*_C_ 83.3 (C-2′), 78.7 (C-3′), 87.2 (C-4′) and 62.9 (C-5′) were similar to those of *α*-l-arabinofuranose in juglanin [16]. In addition, the structure of the sugar part in 12 was further confirmed to be L-arabinofuranose after derivatization and comparison with an authentic sample in a GC analysis. Based on the above evidence and the 2D NMR spectra (Figure 6), the structure of **12** was elucidated to be 3,5,7-trihydroxylchromone-3-*O*-*α*-l-arabinofuranoside.

**Figure 7:**
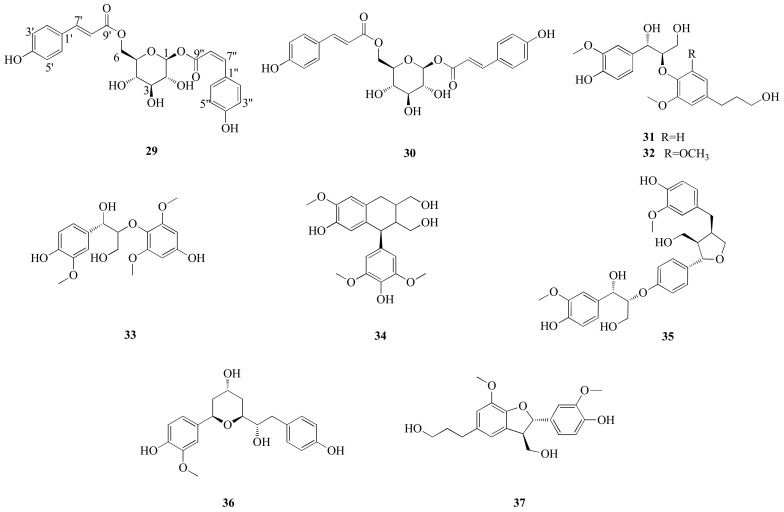
The structures of phenylpropanoids (**29**–**37**) isolated from Diaphragma juglandis Fructus.

Compound **29**, a yellow amorphous powder (Figure 7), had a molecular formula of C_24_H_24_O_10_ based on HRESIMS at *m/z* 471.1286 [M-H]^-^ (calcd for 471.1290) and corresponding to 13 degrees of unsaturation. A comparison of the ^1^H- and ^13^C-NMR spectroscopic data of **29** (Table 3) with those of 1,6-di-*O*-(*E*)-coumaroyl-*β*-d-glucopyranoside (**30**) suggested that these two compounds were closely related in structure [17], while the variations mainly occurred in the vicinity of C-7″ and C-8″ [C-3″/5″ (Δ*δ*_C_ −1.1), C-4″ (−1.3), C-9″ (−2.8), C-2″/6″ (+ 2.8) and C-8″ (+ 0.5)], indicating that 29 was the C-7″/C-8″ *cis-trans* isomerization of 30. The structure of 29 was further confirmed by analysis of coupling constants (*J*_C-7″_ = *J*_C-8″_ = 12.8 Hz in **29** and *J*_C-7″_ = *J*_C-8″_ = 15.9 Hz in **30**) and the 2D NMR spectra (Figure 6). Accordingly, the structure of **29** was characterized as 1-*O*-(*Z*)-coumaroyl,6-*O*-(*E*)-coumaroyl-*β*-d-glucopyranoside.

In addition, 8 known megastigmanes (blumenol B (**4**) [18], vomifoliol (**5**) [19], aglycone of euodionoside G (**6**) [20], bridelionol C (**7**) [21], myrsinionoside A (**8**) [22], byzantionoside B (**9**) [23], blumenol C glucoside (**10**) [23] and (6*R*, 9*S*)-6′-(4″-hydroxybenzoyl)-roseoside (**11**) [24]); 16 known flavonoids, including 5 flavanonols (taxifolin (**13**) [25] and derivatives {taxifolin-3-*β*-d-xylopyranoside (**14**) [26], taxifolin-3-*O*-*α*-l-arabinofuranoside (**15**) [27]}, (+)-catechin (**16**) [28] and derivative {catechin lactone A (**17**) [29]}), 3 flavanones (naringenin derivatives {naringenin 7-*O*-*β*-d-glucopyranoside (**18**) [30]}, eriodictyol derivatives {sakuranetin 5-*O*-*β*-d xylopyranoside (**19**) [30] and (2*R*)-eriodictyol-5-*O*-*β*-d-glucoside (**20**) [31]}), 7 flavonols (quercetin (**21**) [32] and derivatives {3-*O*-methylquercetin (**22**) [33], avicularin (**23**) [34], quercetin-3-*O*-*α*-d-arabinofuranoside (**24**) [34], quercetin 3-*O*-*β*-d-xylopyranoside (**25**) [35], quercetin-3-*O*-(6″-*O*-galloyl)-*β*-d-galactopyranoside (**26**) [36] and quercetin-3-*O*-*β*-d-glucopyranoside (**27**) [37]}) and 1 flavone (luteolin (**28**) [38]); and 8 known phenylpropanoids, including 1 phenylpropionic acid (1,6-di-*O*-(*E*)-coumaroyl-*β*-d-glucopyranoside (**30**) [17]), 3 phenylpropanols (erythro-(7*S*,8*R*)-guaiacyl-glycerol-*β*-*O*-4′-dihydroconiferyl ether (**31**) [39], 1-(4′-hydroxy-3′-methoxyphenyl)-2-[4″-(3-hydroxypropyl)-2″,6″-dimethoxyphenoxy]propane-1,3-diol (**32**) [40] and rosalaevin B (**33**) [41]), 1 cyclolignan (5-methoxy-(+)-isolariciresinol (**34**) [42]), 2 monoepoxylignans (erythro-guaiacyl-glycerol-*β*-*O*-4′-(5′)-methoxylariciresinol (**35**) [43] and rhoiptelol B (**36**) [44] and 1 benzofuran lignan (dihydrodehydodiconiferyl alcohol (**37**) [45]), were isolated from Diaphragma juglandis Fructus and their structures were determined based on the spectroscopic data and the literature (Figure 1, Figure 5 and Figure 7).

### 2.2. Glucosidase Inhibitory Assay

Alpha-glucosidase is an enzyme that hydrolyzes the carbohydrates to monosaccharides in the final step of carbohydrate digestion. Therefore, inhibiting the activity of *α*-glucosidase can effectively inhibit sugar uptake, thereby achieving lowered blood sugar [46]. Diaphragma juglandis Fructus was found to improve symptoms of diabetes [8] and the total flavonoids from it showed significant α-glucosidase inhibitory activities [47]. In order to search for bioactive substances to treat type 2 diabetes using Diaphragma juglandis Fructus, all isolated constituents (**1**–**37**) were assessed for antidiabetic activity using an in vitro α-glycosidase inhibition assay. As shown in Table 4, compounds **13, 16, 21** and **28** exhibited much more potent activity with the IC_50_ values of 40.39, 54.82, 29.47 and 35.41 µM being lower than the positive control, acarbose (60.01 µM). However, the IC_50_ values of other compounds were either over the positive control or inactive for α-glycosidase inhibition at 100 µM.

Most of the bioactive compounds were flavonoids, suggesting that flavonoids might be the main bioactive substances contributing to the α-glucosidase inhibitory activity of Diaphragma juglandis Fructus. Furthermore, the structures of the A, B and C rings in the flavonoids were closely related to the inhibitory activity. Consistent with the previous reports, comparison among quercetin (**21**) and luteolin (**28**) revealed that hydroxylation at the 3-position of flavone enhanced the inhibitory effect. Comparison among (+)-catechin (**16**), quercetin (**21**) and luteolin (**28**) suggested the saturation of the 2,3-double bond in the C ring seemed to decrease the inhibitory activity [48]. In addition, among the taxifolin (**13**) and derivatives (**14, 15**), taxifolin showed the strongest inhibitory effect, indicating that the presence of a sugar moiety at C-3 may be responsible for the lowered activity [49]. In addition, galloyl moieties strengthen the inhibitory effects of flavonoids against the α-glucosidase (such as compound **26**) [50].

## 3. Materials and Methods

### 3.1. General Experimental Procedures

Optical rotations were determined by a Jasco P-2000 digital polarimeter. CD spectra were recorded on a Bio-Logic MOS-450 spectropolarimeter. One-dimensional (1D) and two-dimensional (2D) NMR spectra were measured on a Bruker 600 spectrometer. HRESIMS data were obtained using a Q Exactive Focus LC-MS/MS spectrometer (Thermo Fisher, MA, USA) or Triple TOF™ 5600 MS/MS system from Applied AB Sciex (Foster City, CA, USA). Medium-pressure liquid chromatography was performed with Buchi C610. To perform the preparative HPLC separation, a C_18_ preparative HPLC colum (21.2 mm × 250 mm, 5 µm, Sharpsil-U C18) on a Shimadzu LC-16P instrument equipped with an RID-20A refractive index detector was used for purification. Silica gel (200–300 mesh, Qingdao Marine Chemical Ltd., Qingdao, China) and RP-18 reversed-phase silica gel (YMC Company Ltd., Tokyo, Japan) were used for column chromatography. All organic solvents were analytical grade (Tianjin zhiyuan Chemical Regents Co., Ltd., Tianjin, China).

The α-glucosidase was obtained from Sigma-Aldrich (Shanghai) Trading Co., Ltd (Shanghai, China). p-nitrophenyl-α-d-glucopyranoside was obtained from Macklin Co., Ltd. (Lot# C12592180, Shanghai, China). Acarbose was purchased from bayer Co., Ltd. (Lot# BJ59027, Beijing, China).

### 3.2. Plant Material

Diaphragma juglandis Fructus was purchased from Anguo Chinese medicinal materials markets (Hebei Province) in March 2021 and identified by Professor Xiang-ping Pei (Shanxi University of Chinese Medicine). A voucher specimen (No. 20210301) was deposited at Shanxi Modern Chinese Medicine Engineering laboratory (Shanxi University of Chinese Medicine).

### 3.3. Extraction and Isolation

Diaphragma juglandis Fructus (5 kg) was extracted with 70% EtOH (40 L × 3) and dried under reduced pressure to afford 1.1 kg of crude extract. This extract was then suspended in H_2_O and partitioned sequentially with petroleum ether (82 g), EtOAc (280 g) and *n*-butyl alcohol (416 g). The EtOAc partition (260 g) was then fractionated on a silica gel column (CH_2_Cl_2_-MeOH, 50:0, 50:1, 30:1, 15:1, 10:1, 5:1, 3:1, 2:1, 1:1, 0:1, *v*/*v*) to yield 8 fractions (A-H). Of these, fraction B (15.3 g) was chromatographed on an ODS column (MeOH-H_2_O, 10–100% MeOH) to yield 15 subfractions (B1-B15). Subfraction B3 was subjected to purification over a preparative HPLC to afford compounds **4** (2.5 mg, *t*_R_ = 16.5 min) and **5** (3.7 mg, *t*_R_ = 16.7 min). B4 was further purified by a preparative HPLC to obtain compounds **13** (4.2 mg, *t*_R_ = 16.8 min) and **34** (1.6 mg, *t*_R_ = 15.6 min). Then, B5 was also purified using a preparative HPLC to give compounds **1** (3.1 mg, *t*_R_ = 18.9 min), **6** (2.6 mg, *t*_R_ = 18.8 min), **31** (2.7 mg, *t*_R_ = 16.5 min) and **32** (6.5 mg, *t*_R_ = 16.9 min). B7 was separated by a preparative HPLC to create compounds **33** (6.5 mg, *t*_R_ = 18.3 min) and **36** (3.9 mg, *t*_R_ = 17.8 min). B8 was purified by a preparative HPLC to yield compounds **35** (12.1 mg, *t*_R_ = 19.8 min) and **37** (32.3 mg, *t*_R_ = 19.2 min). B9 was applied to a preparative HPLC to give compounds **29** (5.2 mg, *t*_R_ = 20.5 min) and **30** (12.8 mg, *t*_R_ = 20.1 min). B11 was purified with a preparative HPLC to yield compounds **19** (4.2 mg, *t*_R_ = 22.2 min), **20** (5.3 mg, *t*_R_ = 23.1 min) and **21** (2.1 mg, *t*_R_ = 23.3 min). Fraction C (20.7 g) was then eluted on an ODS column to yield 13 subfractions (C1-C13). One of these subfractions, C2, was further purified on a preparative HPLC. As a result, crops of compounds **2** (2.9 mg, *t*_R_ = 13.5 min), **7** (1.4 mg, *t*_R_ = 14.0 min), **12** (15.8 mg, *t*_R_ = 14.8 min) and **27** (8.0 mg, *t*_R_ = 11.5 min) were obtained. Two other subfractions (C8 and C10) were further applied to a preparative HPLC to afford compounds **8** (6.3 mg, *t*_R_ = 20.9 min) and **3** (2.8 mg, *t*_R_ = 22.9 min). On the other hand, fraction D (10.8 g) was eluted on an ODS column (MeOH-H_2_O, 10–100% MeOH) to obtain 12 subfractions (D1-D12). Subfraction D6 was purified using a preparative HPLC to give compounds **9** (9.0 mg, *t*_R_ = 19.9 min), **10** (13.9 mg, *t*_R_ = 20.0 min), **11** (10.8 mg, *t*_R_ = 19.5 min), **15** (5.1 mg, *t*_R_ = 18.8 min), **22** (20.6 mg, *t*_R_ = 19.2 min) and **23** (2.9 mg, *t*_R_ = 19.6 min). Fraction E (13.5 g) was separated on an ODS column (MeOH-H_2_O, 10–100% MeOH) to obtain 10 subfractions (E1-E10). Subfraction E7 was purified using a preparative HPLC to yield compounds **14** (8.2 mg, *t*_R_ = 16.0 min) and **18** (23.9 mg, *t*_R_ = 17.9 min). Then, fraction F (18.5 g) was chromatographed on an ODS column (MeOH-H_2_O, 10–100% MeOH) to give 11 subfractions (F1-F11), and F7 was separated on a preparative HPLC. As a result, compounds **16** (10.1 mg, *t*_R_ = 16.3 min), 1**7** (7.5 mg, *t*_R_ = 16.8 min), **25** (21.6 mg, *t*_R_ = 17.5 min) and **26** (7.5 mg, *t*_R_ = 18.6 min) were obtained. F8 was purified with a preparative HPLC to obtain compounds **24** (11.8 mg, *t*_R_ = 19.0 min) and **28** (2.8 mg, *t*_R_ = 18.7 min).

### 3.4. Characterization of Compounds ***1***–***3***, ***12***, ***29***

Diamegastigmane A (**1**): white amorphous powder; ${\left[\alpha \right]}_{D}^{20}$ = +11 (c = 0.1, MeOH); HRESIMS *m/z* 225.1489 [M+H]^+^ (calcd. for C_13_H_21_O_3_, *m/z* 225.1490); ^1^H-NMR (methanol-*d*_4_, 600 MHz) and ^13^C-NMR (methanol-*d*_4_, 150 MHz), see Table 1.

Diamegastigmane B (**2**): white amorphous powder; ${\left[\alpha \right]}_{D}^{20}$ = +10 (c = 0.1, MeOH); HRESIMS *m/z* 485.3115 [2M+H]^+^ (calcd. for C_26_H_45_O_8_, *m/z* 485.3114); ^1^H-NMR (methanol-*d*_4_, 600 MHz) and ^13^C-NMR (methanol-*d*_4_, 150 MHz), see Table 1.

Diamegastigmane C (**3**): white amorphous powder; ${\left[\alpha \right]}_{D}^{20}$ = +35 (c = 0.1, MeOH); HRESIMS *m/z* 493.2442 [M+H]^+^ (calcd. for C_26_H_37_O_9_, *m/z* 493.2437); ^1^H-NMR (methanol-*d*_4_, 600 MHz) and ^13^C-NMR (methanol-*d*_4_, 150 MHz), see Table 1.

3,5,7-trihydroxylchromone-3-*O*-*α*-l-arabinofuranoside (**12**): yellow amorphous powder; ${\left[\alpha \right]}_{D}^{20}$ = -166 (c = 0.1, MeOH); HRESIMS *m/z* 327.0715 [M+H]^+^ (calcd. for C_14_H_15_O_9_, *m/z* 327.0716); ^1^H-NMR (methanol-*d*_4_, 600 MHz) and ^13^C-NMR (methanol-*d*_4_, 150 MHz), see Table 2.

1-*O*-(*Z*)-coumaroyl,6-*O*-(*E*)-coumaroyl-*β*-d-glucopyranoside (**29**): Yellow amorphous powder; ${\left[\alpha \right]}_{D}^{24}$ = -50 (c = 0.3, MeOH); HRESIMS *m/z* 471.1286 [M-H]^-^ (calcd. for C_24_H_23_O_10_, *m/z* 471.1290); ^1^H-NMR (methanol-*d*_4_, 600 MHz) and ^13^C-NMR (methanol-*d*_4_, 150 MHz), see Table 3.

### 3.5. Acid Hydrolysis of Compounds ***3***, ***12*** and ***29***

In order to determine the absolute configuration of monosaccharides in the new compounds, the acid hydrolysis of compounds **3**, **12** and **29** were performed according to the previous literature [51]. The *n*-hexane fractions were then detected by GC-MS with a DB-5 capillary column, and the absolute configuration of sugar components was confirmed to be D-glucose, L-arabinofuranose and d-glucose in compounds **3**, **12** and **29**, respectively, compared with standards.

### 3.6. α-Glucosidase Inhibitory Assay

The α-glucosidase inhibition assay was performed according to a previous report [52] with a slight difference: the concentration of α-glucosidase was diluted to 0.15 unit/mL. The volume of α-glucosidase added to the 96-well plate was 10 μL. Acarbose was used as the positive control.

## 4. Conclusions

In summary, a detailed phytochemical investigation on the EtOAc partition of 70% ethanol extract of Diaphragma juglandis Fructus was carried out to afford 37 constituents in this research, and five of them were new structures (**1**–**3**, **12**, **29**). Their structures were elucidated based on MS and NMR spectroscopic data and comparison with data reported in the literature. Compounds **1**–**3** were new megastigmanes. Among them, compound **1** was a bicyclic neomegastigmane, and a plausible biogenetic pathway for it was further discussed in this paper. Compound **12** was a new chromone with α-l-arabinofuranoside. Compound **29** was a new phenylpropanoid. The α-glucosidase inhibition activity was also investigated. Compounds **13**, **16**, **21** and **28** were found to be quite potent and most of them were flavonoids, suggesting that flavonoids might be the main bioactive substances contributing to the α-glucosidase inhibitory activity of Diaphragma juglandis Fructus. These findings also revealed that these compounds could be target compounds for the development of new antidiabetic agents.

## Figures and Tables

**Table 1:** ^1^H-NMR (600 MHz, methanol-*d*_4_) and ^13^C-NMR (150 MHz, methanol-*d*_4_) of compounds **1**–**3** (*δ* in ppm, *J* values in Hz).

Position	1		2		3	
	*δ*_H_	*δ*_C_	*δ*_H_	*δ*_C_	*δ*_H_	*δ*_C_
1	-	35.5	-	43.1	-	37.2
2	2.33 (d, 15.6) 2.13 (d, 15.6)	50.4	2.61 (d, 18.3) 2.20 (d, 18.3)	50.8	2.34 (d, 17.3) 1.87 (d, 17.3)	48.1
3	-	202.6	-	200.9	-	202.3
4	6.15 (br. s)	121.0	6.10 (br. s)	122.4	5.70 (br. s)	125.3
5	-	168.2	-	173.9	-	169.9
6	2.16 (dd, 12.9, 4.5)	49.2	-	78.8	1.81 (t, 5.2)	52.2
7	1.94 (m) 1.39 (dd, 13.4, 4.0)	25.3	1.98 (m) 1.78 (m)	35.8	1.92 (m) 1.35 (m)	26.8
8	1.86 (ddd, 13.4, 3.7, 3.0) 1.72 (td, 13.4, 4.3)	39.1	1.67 (m) 1.43 (m)	34.9	2.19 (m) 1.55 (m)	37.6
9	-	76.8	3.67 (m)	68.9	3.73 (m)	76.5
10	1.03 (s)	19.3	1.16 (d, 6.2)	23.5	1.17 (d, 6.2)	20.4
11	1.00 (s)	25.8	1.09 (s)	24.4	0.90 (s)	28.9
12	1.05 (s)	28.7	1.04 (s)	23.5	0.96 (s)	27.4
13	4.08 (d, 1.4)	80.2	4.39 (t, 1.9)	63.1	1.90 (d, 1.1)	24.8
1′					4.34 (d, 7.7)	102.8
2′					3.17 (m)	75.2
3′					3.39 (m)	78.0
4′					3.39 (m)	72.0
5′					3.58 (m)	75.2
6′					4.56 (dd, 11.8, 2.3) 4.42 (dd, 11.8, 6.4)	64.9
1″					-	122.3
2″, 6″					7.89 (d, 8.8)	132.9
3″, 5″					6.83 (d, 8.8)	116.2
4″					-	163.7
7″					-	167.9

“m” means multiplet or overlapped with other signals.

**Table 2:** ^1^H-NMR (600 MHz, methanol-*d*_4_) and ^13^C-NMR (150 MHz, methanol-*d*_4_) of compound **12**. (*δ* in ppm, *J* values in Hz).

Position	*δ*H	*δ*C	Position	*δ*H	*δ*C
2	8.09 (s)	148.7	9	-	159.3
3	-	140.0	10	1.03 (s)	106.4
4	-	179.0	1′	5.48 (s)	109.5
5	-	163.4	2′	4.31 (dd, 3.2, 1.1)	83.3
6	6.20 (dd, 2.1)	100.0	3′	3.94 (dd, 5.9, 3.2)	78.7
7	-	166.1	4′	4.13 (m)	87.2
8	6.31 (d, 2.1)	95.0	5′	3.78 (dd, 12.1, 3.4) 3.67 (dd, 12.1, 5.6)	62.9

“m” means multiplet or overlapped with other signals.

**Table 3:** ^1^H-NMR (600 MHz) and ^13^C-NMR (150 MHz) of **29** in methanol-*d*_4_ (*δ* in ppm, *J* values in Hz).

Position	*δ*H	*δ*C	Position	*δ*H	*δ*C
1	5.57 (d, 8.2)	95.5	7′	7.64 (d, 15.9)	146.9
2	3.39 (m)	73.9	8′	6.37 (d, 15.9)	114.9
3	3.48 (m)	77.9	9′	-	169.1
4	3.42 (m)	71.3	1″	-	127.4
5	3.67 (m)	76.3	2″,6″	7.71 (d, 8.7)	134.1
6	4.52 (dd, 12.0, 1.9) 4.32 (dd, 12.0, 5.5)	64.4	3″,5″	6.75 (d, 8.7)	115.9
1′	-	127.2	4″	-	160.4
2′,6′	7.45 (d, 8.6)	131.2	7″	6.95 (d, 12.8)	147.0
3′,5′	6.79 (d, 8.6)	116.8	8″	5.83 (d, 12.8)	115.4
4′	-	161.3	9″	-	166.5

“m” means multiplet or overlapped with other signals.

**Table 4:** α-Glucosidase inhibitory activity of compounds **1**–**37**.

Compound	IC_50_ (µM)	Compound	IC_50_ (µM)
**1–11**	>100	**22**	67.74 ± 6.41
**12**	92.35 ± 7.24	**23–25,27**	>100
**13**	40.39 ± 4.14	**26**	77.15 ± 12.36
**14**	95.78 ± 12.62	**28**	35.41 ± 3.87
**15, 17–20**	>100	**29–34,36,37**	>100
**16**	54.82 ± 7.47	**35**	87.74 ± 13.41
**21**	29.47 ± 2.95	**Acarbose**	60.01 ± 4.82

Data expressed as mean ± SD (*n* = 3).

## Data Availability

Not applicable.

## Supplementary Materials

The following supporting information can be downloaded at: https://www.mdpi.com/article/10.3390/molecules27103045/s1. A scheme of extraction and isolation (Figure S1), spectroscopic data for compounds **1**–**3**, **12** and **29** (Figures S2–S39), ^13^C-NMR spectra of compounds **4**–**11**, **13**–**28**, **30**–**37** (Figures S40–S71) are available as Supporting Information.

## Sample Availability

Samples of all the isolated compounds are available from the authors.
