# Supplementary material for: The Chemical Constituents of Diaphragma Juglandis Fructus and Their Inhibitory Effect on α-Glucosidase Activity
Source: Molecules. 2022 May 10;27(10):3045. doi: 10.3390/molecules27103045 (PMC9143591; doi:10.3390/molecules27103045)
Supplement: Supplementary file 1 [file molecules-27-03045-s001.zip › molecules-1686623-supplementary.pdf]

SUPPLEMENTARY MATERIAL

Chemical Constituents of Diaphragma juglandis Fructus and Their Inhibitory Effect on α-Glucosidase Activity

Jinyan Tan[1,†], Yangang Cheng[1,2,†], Shihui Wang[1], Jianli Li[1], Haiqin Ren[1], Yuanbiao Qiao[2], Qingshan Li[2,*], Yingli Wang[1,2,*]

1. Shanxi Modern Chinese Medicine Engineering Laboratory, Shanxi University of Chinese Medicine, Jinzhong 030619, China

2. Shanxi Key Laboratory of Innovative Drug for the Treatment of Serious Diseases Basing on the Chronic Inflammation, Shanxi University of Chinese Medicine, Jinzhong 030619, China

* Correspondence: wyltyut@163.com (Y.W.); sxlqs0501@sxtcm.edu.cn (Q.L.)

† These authors contributed equally to this work.

Contents

Figure S1. Scheme of extraction and isolation.

Figure S2. HR-ESI-MS spectrum of **1**.

Figure S3. ^{1}H NMR spectrum of **1** (600 MHz, CD_3OD).

Figure S4. ^{13}C NMR spectrum of **1** (150 MHz, CD_3OD).

Figure S5. DEPT 135 spectrum of **1**.

Figure S6. HSQC spectrum of **1**.

Figure S7. ^{1}H-^{1}H COSY spectrum of **1**.

Figure S8. HMBC spectrum of **1**.

Figure S9. NOESY spectrum of **1**.

Figure S10. HR-ESI-MS spectrum of **2**.

Figure S11. ^{1}H NMR spectrum of **2** (600 MHz, CD_3OD).

Figure S12. ^{13}C NMR spectrum of **2** (150 MHz, CD_3OD).

Figure S13. DEPT 135 spectrum of **2**.

Figure S14. HSQC spectrum of **2**.

Figure S15. ^{1}H-^{1}H COSY spectrum of **2**.

Figure S16. HMBC spectrum of **2**.

Figure S17. CD spectrum of **2**.

Figure S18. HR-ESI-MS spectrum of **3**.

Figure S19. ^{1}H NMR spectrum of **3** (600 MHz, CD_3OD).

Figure S20. ^{13}C NMR spectrum of **3** (150 MHz, CD_3OD).

Figure S21. DEPT 135 spectrum of **3**.

Figure S22. HSQC spectrum of **3**.

Figure S23. ^{1}H-^{1}H COSY spectrum of **3**.

Figure S24. HMBC spectrum of **3**.

Figure S25. CD spectrum of **3**.

Figure S26. HR-ESI-MS spectrum of **12**.

Figure S27. ^{1}H NMR spectrum of **12** (600 MHz, CD_3OD).

Figure S28. ^{13}C NMR spectrum of **12** (150 MHz, CD_3OD).

Figure S29. DEPT 135 spectrum of **12**.

Figure S30. HSQC spectrum of **12**.

Figure S31. ^{1}H-^{1}H COSY spectrum of **12**.

Figure S32. HMBC spectrum of **12**.

Figure S33. HR-ESI-MS spectrum of **29**.

Figure S34. ^{1}H NMR spectrum of **29** (600 MHz, CD_3OD).

Figure S35. ^{13}C NMR spectrum of **29** (150 MHz, CD_3OD).

Figure S36. DEPT 135 spectrum of **29**.

Figure S37. HSQC spectrum of **29**.

Figure S38. ^{1}H-^{1}H COSY spectrum of **29**.

Figure S39. HMBC spectrum of **29**.

Figure S40 ^{13}C NMR spectrum of **4** (150 MHz, CD$_3$OD).

Figure S41 ^{13}C NMR spectrum of **5** (150 MHz, CD$_3$OD).

Figure S42 ^{13}C NMR spectrum of **6** (150 MHz, CD$_3$OD).

Figure S43 ^{13}C NMR spectrum of **7** (150 MHz, CD$_3$OD).

Figure S44 ^{13}C NMR spectrum of **8** (150 MHz, CD$_3$OD).

Figure S45 ^{13}C NMR spectrum of **9** (150 MHz, CD$_3$OD).

Figure S46 ^{13}C NMR spectrum of **10** (150 MHz, CD$_3$OD).

Figure S47 ^{13}C NMR spectrum of **11** (150 MHz, CD$_3$OD).

Figure S48 ^{13}C NMR spectrum of **13** (150 MHz, CD$_3$OD).

Figure S49 ^{13}C NMR spectrum of **14** (150 MHz, CD$_3$OD).

Figure S50 ^{13}C NMR spectrum of **15** (150 MHz, CD$_3$OD).

Figure S51 ^{13}C NMR spectrum of **16** (150 MHz, CD$_3$OD).

Figure S52 ^{13}C NMR spectrum of **17** (150 MHz, CD$_3$OD).

Figure S53 ^{13}C NMR spectrum of **18** (150 MHz, CD$_3$OD).

Figure S54 ^{13}C NMR spectrum of **19** (150 MHz, CD$_3$OD).

Figure S55 ^{13}C NMR spectrum of **20** (150 MHz, CD$_3$OD).

Figure S56 ^{13}C NMR spectrum of **21** (150 MHz, CD$_3$OD).

Figure S57 ^{13}C NMR spectrum of **22** (150 MHz, CD$_3$OD).

Figure S58 ^{13}C NMR spectrum of **23** (150 MHz, CD$_3$OD).

Figure S59 ^{13}C NMR spectrum of **24** (150 MHz, CD$_3$OD).

Figure S60 ^{13}C NMR spectrum of **25** (150 MHz, CD$_3$OD).

Figure S61 ^{13}C NMR spectrum of **26** (150 MHz, CD$_3$OD).

Figure S62 ^{13}C NMR spectrum of **27** (150 MHz, CD$_3$OD).

Figure S63 ^{13}C NMR spectrum of **28** (150 MHz, CD$_3$OD).

Figure S64 ^{13}C NMR spectrum of **30** (100 MHz, CD$_3$OD).

Figure S65 ^{13}C NMR spectrum of **31** (150 MHz, CD$_3$OD).

Figure S66 ^{13}C NMR spectrum of **32** (150 MHz, CD$_3$OD).

Figure S67 ^{13}C NMR spectrum of **33** (150 MHz, CD$_3$OD).

Figure S68 ^{13}C NMR spectrum of **34** (150 MHz, CD$_3$OD).

Figure S69 ^{13}C NMR spectrum of **35** (150 MHz, CD$_3$OD).

Figure S70 ^{13}C NMR spectrum of **36** (150 MHz, CD$_3$OD).

Figure S71 ^{13}C NMR spectrum of **37** (150 MHz, CD$_3$OD).

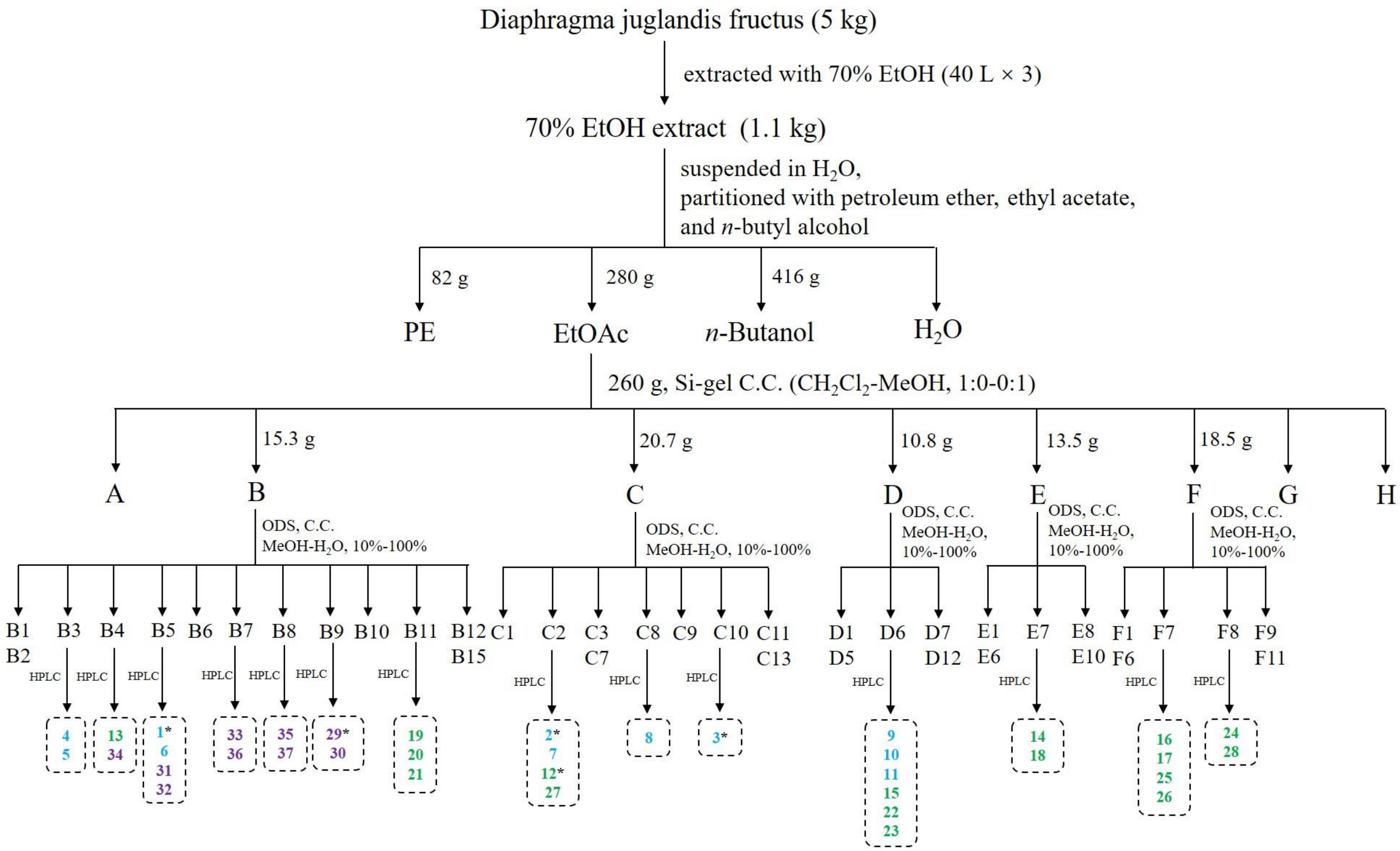

(*: new compounds; blue: megastigmanes; green: flavonoids; purple: phenylpropanoids)

Figure S1. Scheme of extraction and isolation.

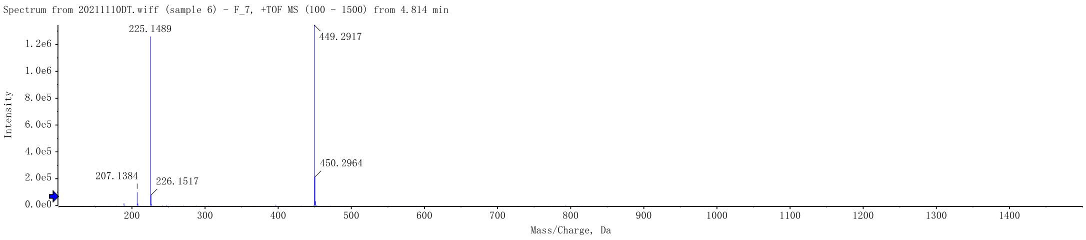

Figure S2. HR-ESI-MS spectrum of **1**

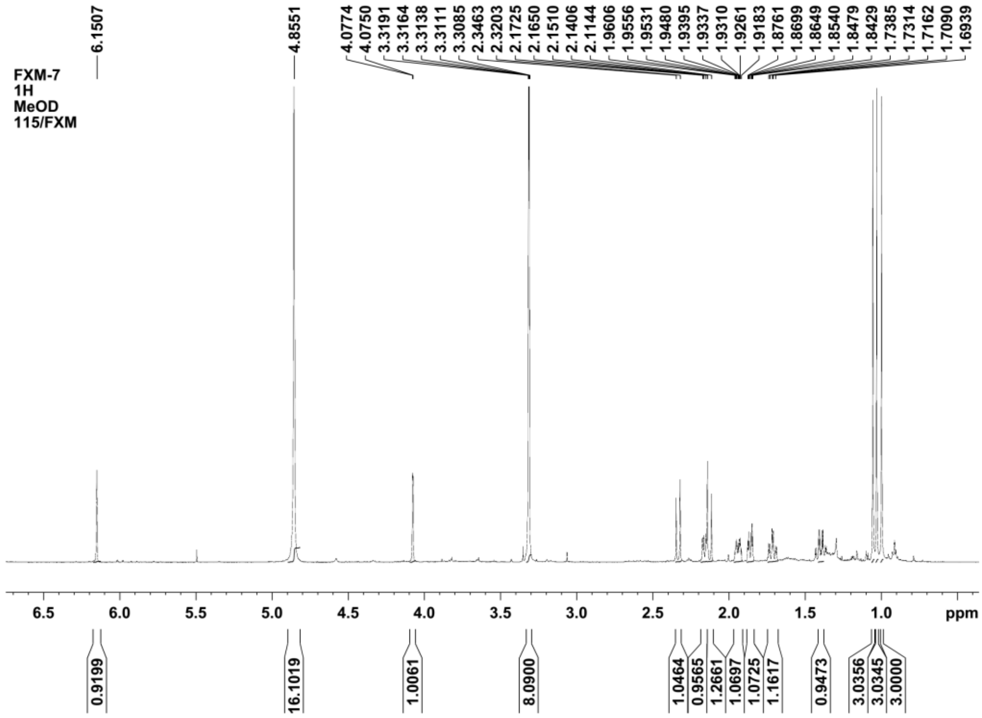

Figure S3. ^{1}H NMR spectrum of **1** (600 MHz, CD_3OD)

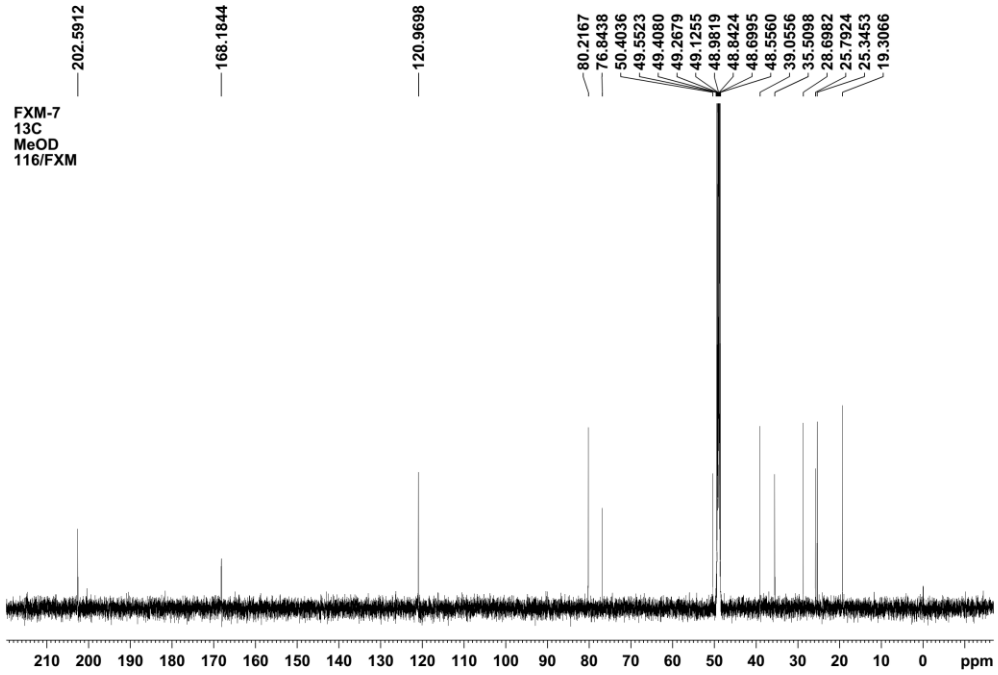

Figure S4. ^{13}C NMR spectrum of **1** (150 MHz, CD_3OD)

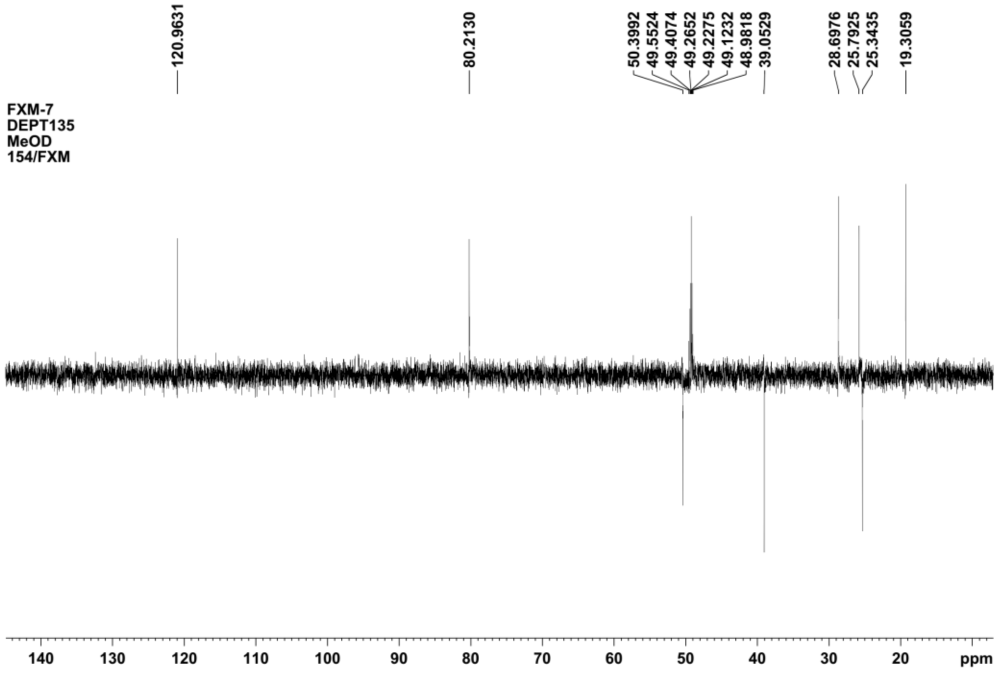

Figure S5. DEPT 135 spectrum of **1**

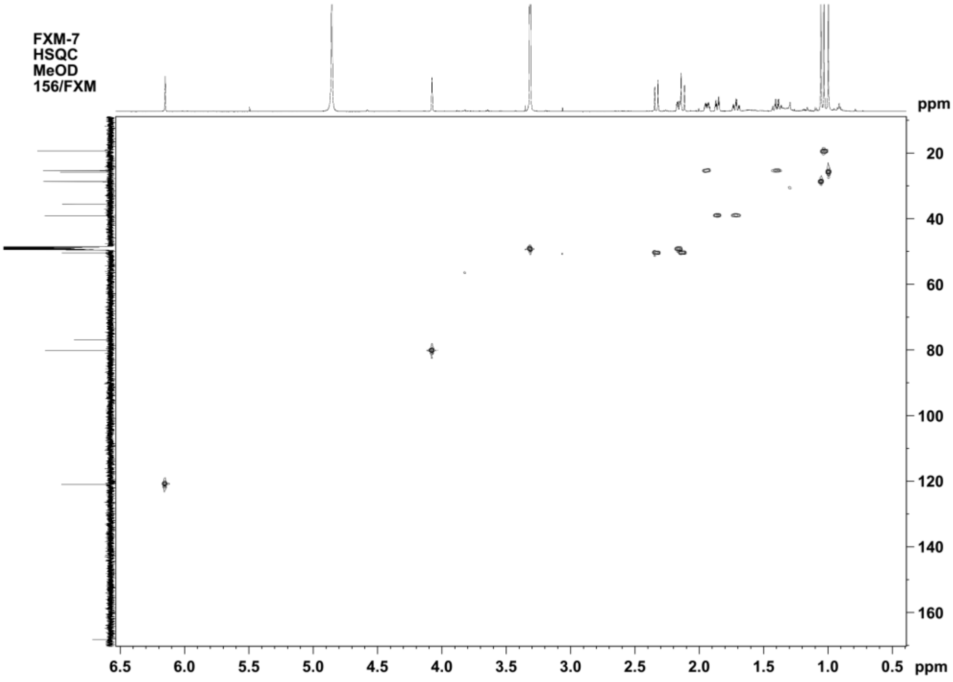

Figure S6. HSQC spectrum of **1**

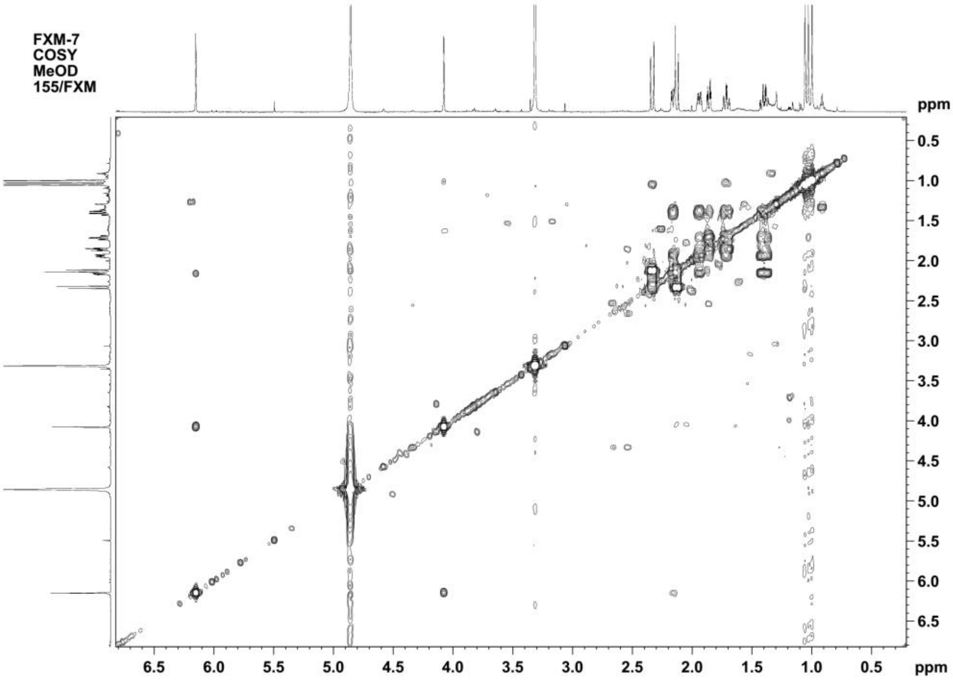

Figure S7. ^{1}H-^{1}H COSY spectrum of **1**

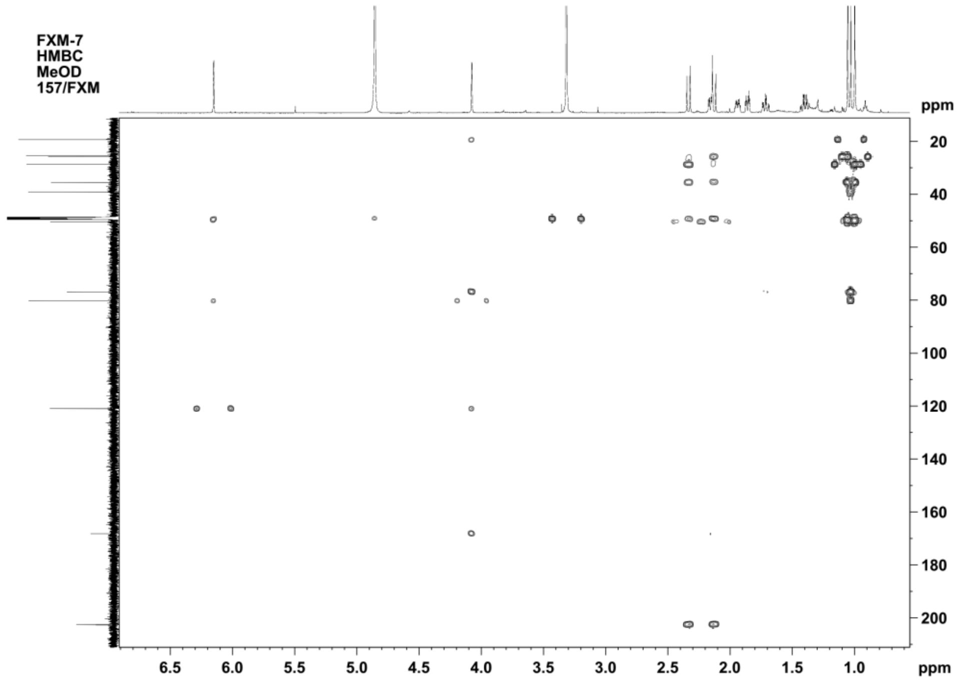

Figure S8. HMBC spectrum of **1**

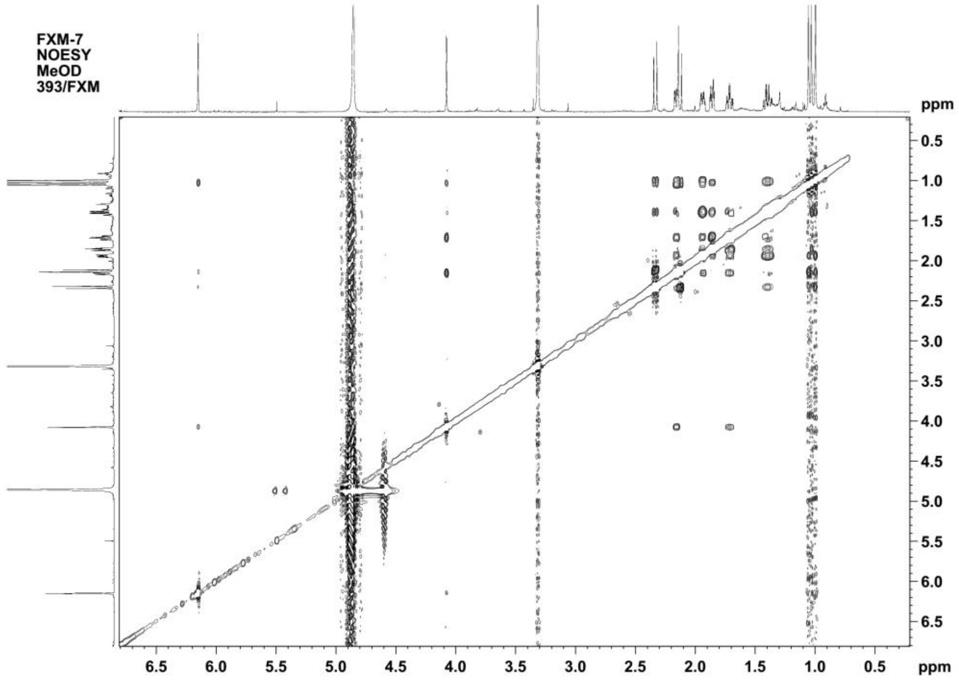

Figure S9. NOESY spectrum of **1**

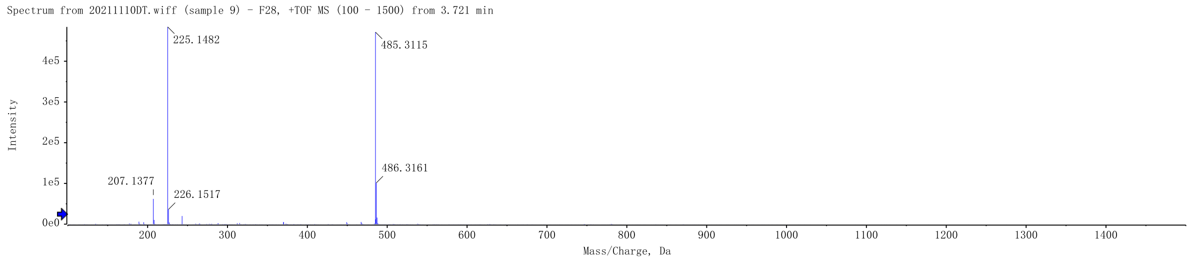

Figure S10. HR-ESI-MS spectrum of **2**

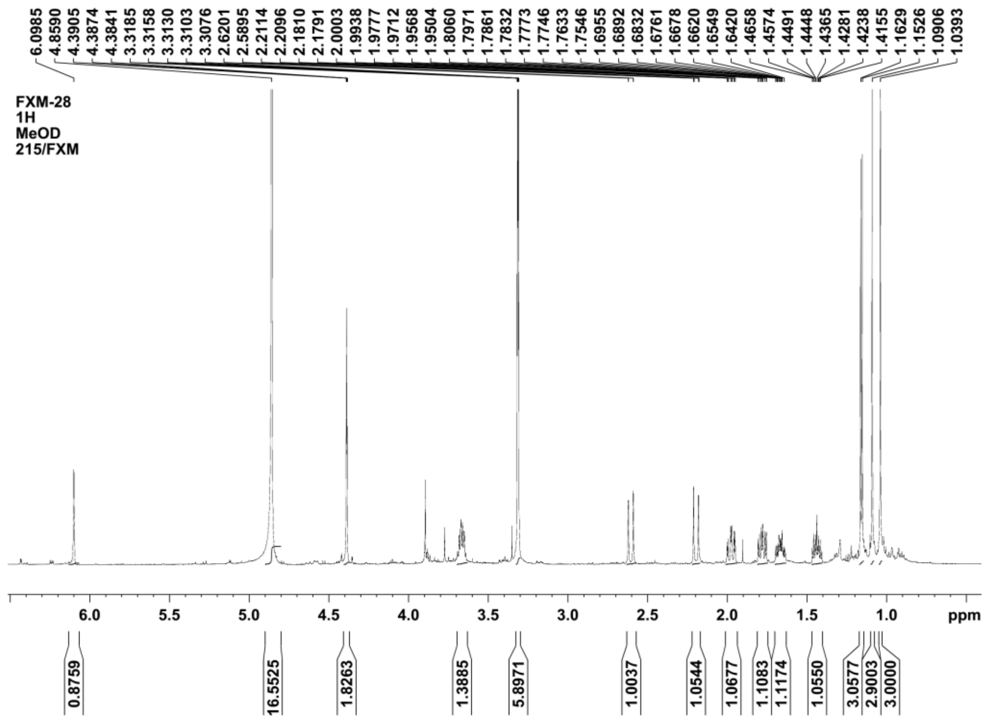

Figure S11. ^{1}H NMR spectrum of **2** (600 MHz, CD_3OD)

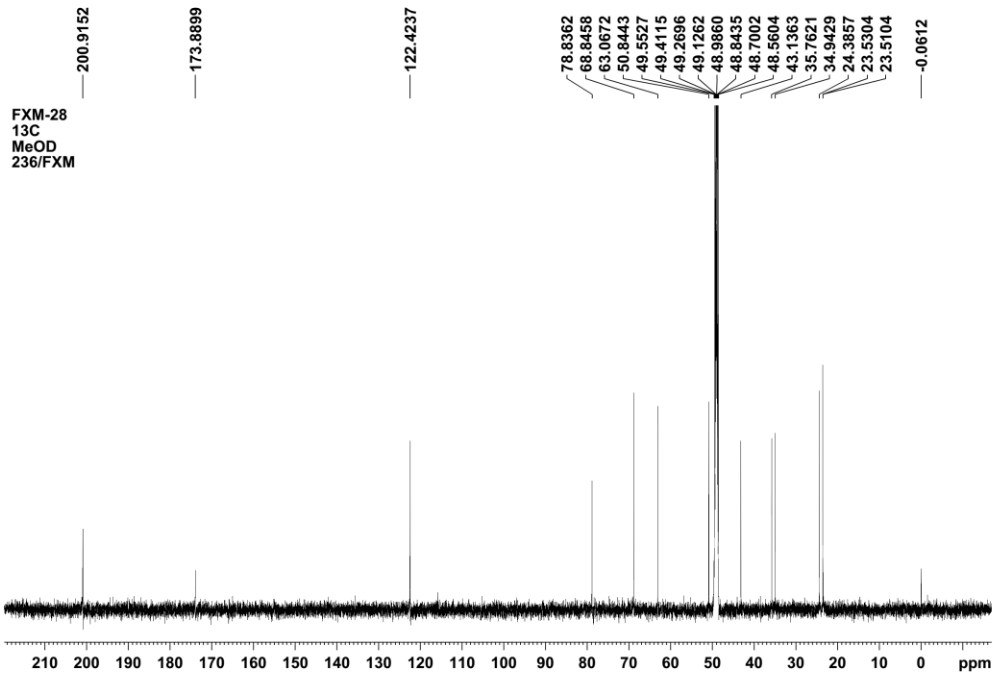

Figure S12. ^{13}C NMR spectrum of **2** (150 MHz, CD_3OD)

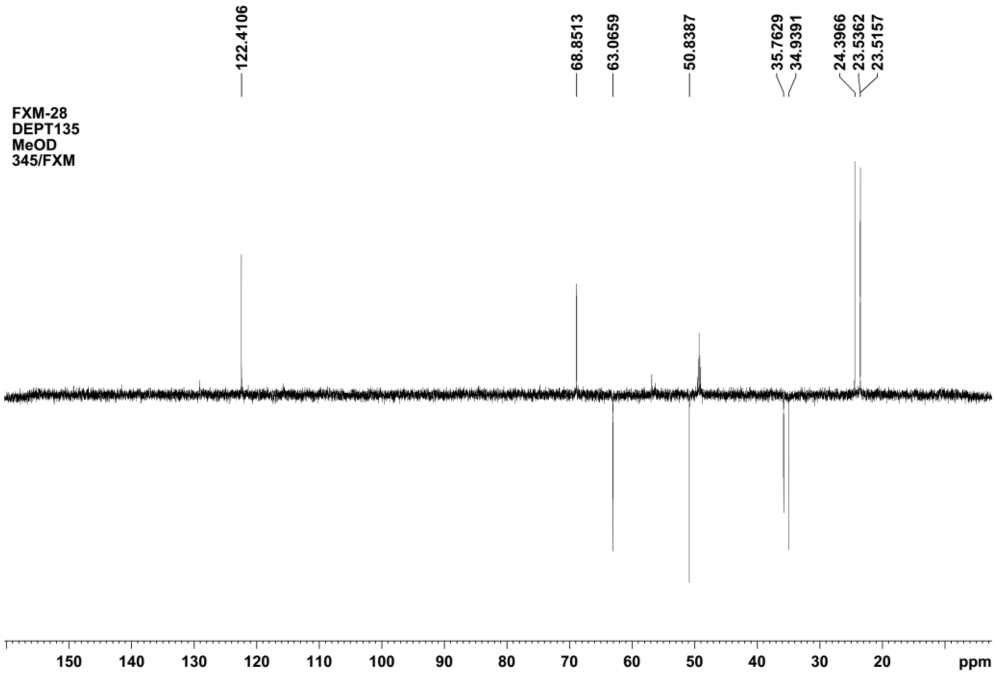

Figure S13. DEPT 135 spectrum of **2**

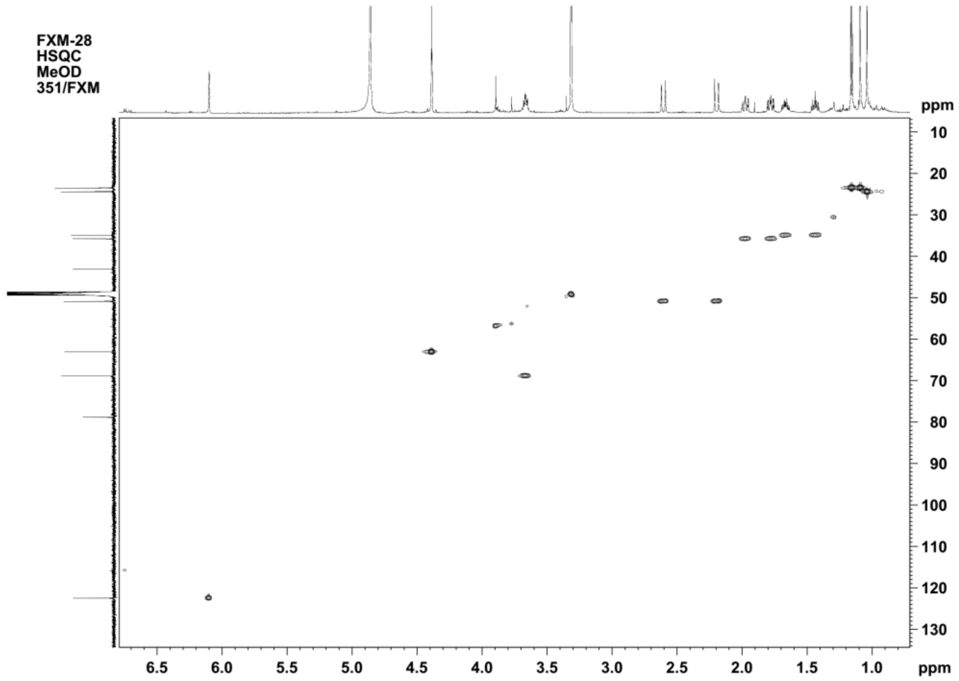

Figure S14. HSQC spectrum of **2**

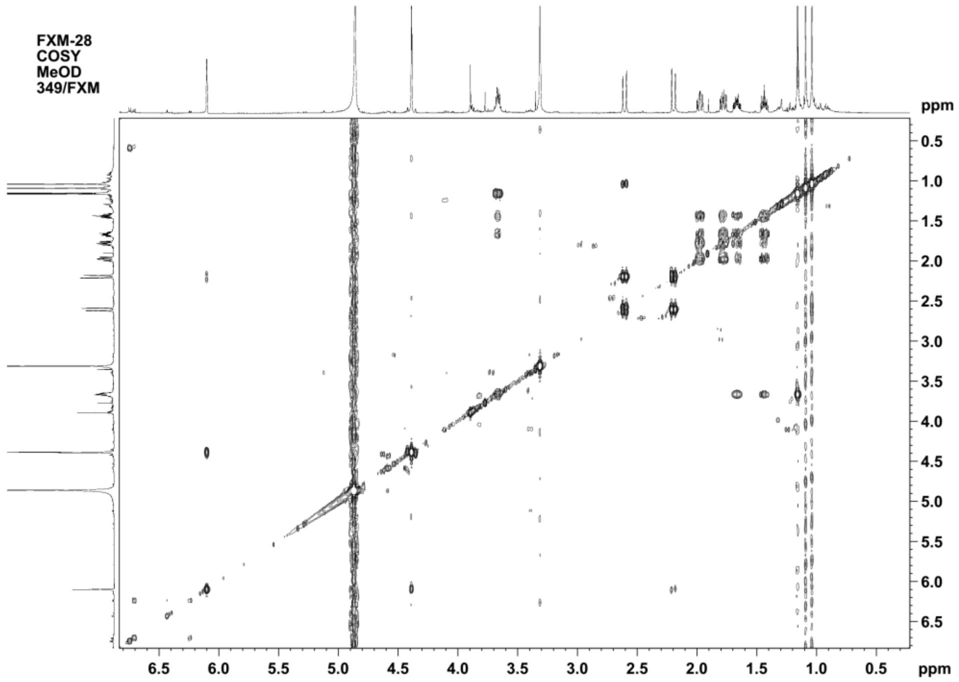

Figure S15. ^{1}H-^{1}H COSY spectrum of **2**

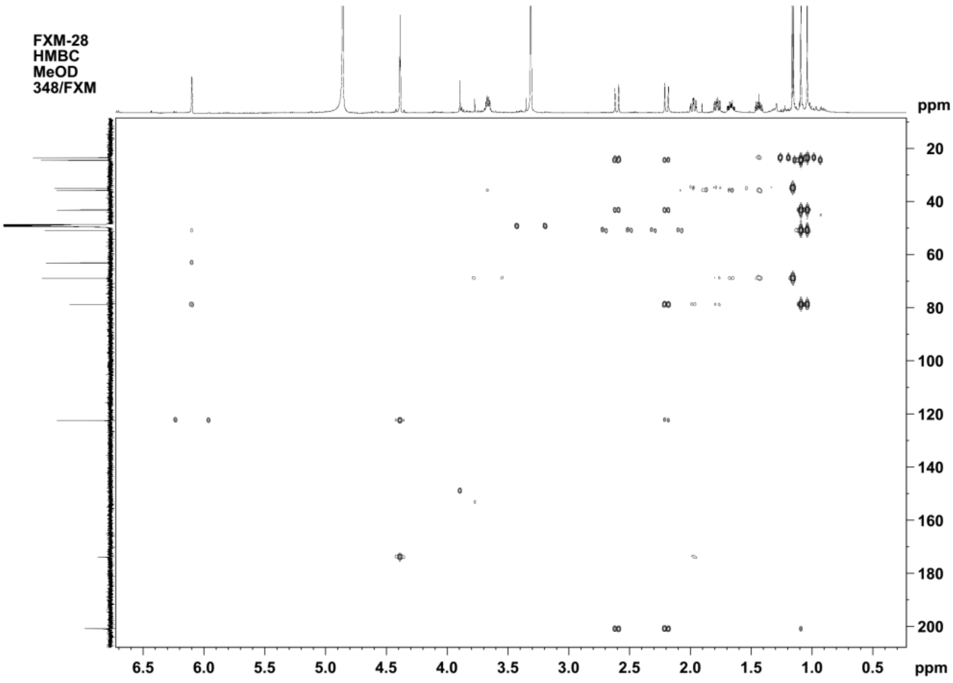

Figure S16. HMBC spectrum of **2**

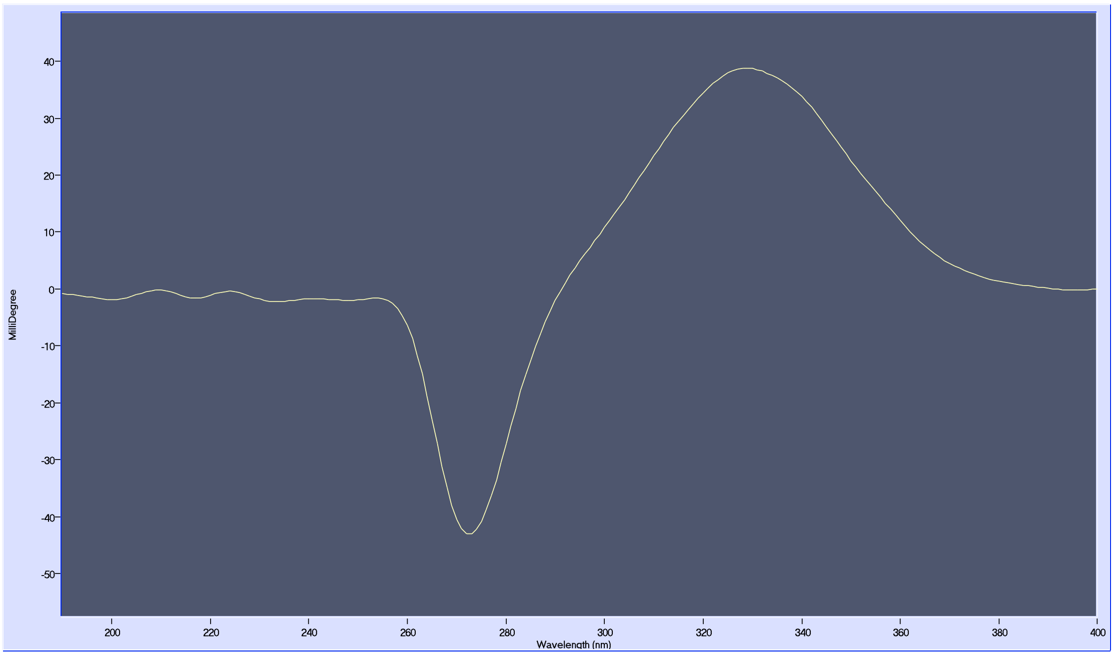

Figure S17. CD spectrum of **2**

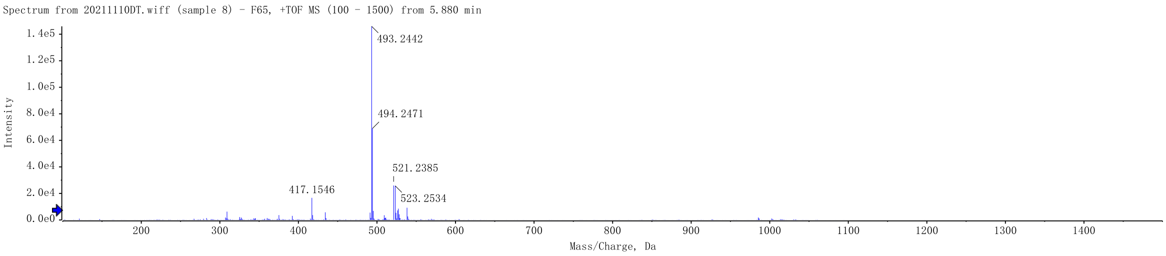

Figure S18. HR-ESI-MS spectrum of **3**

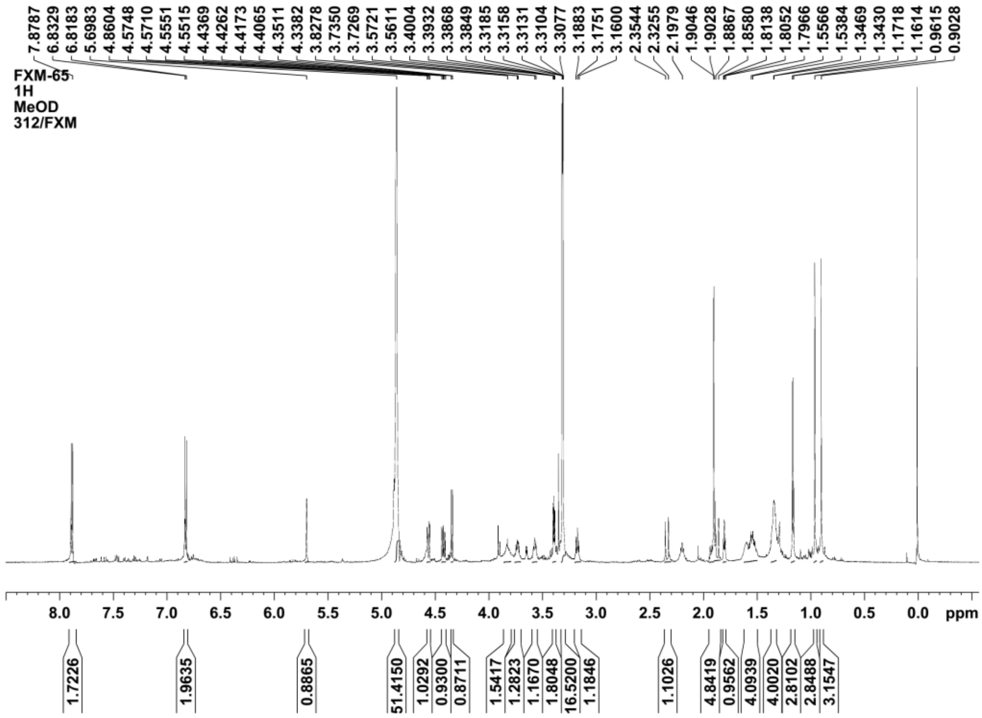

Figure S19. ^{1}H NMR spectrum of **3** (600 MHz, CD_3OD)

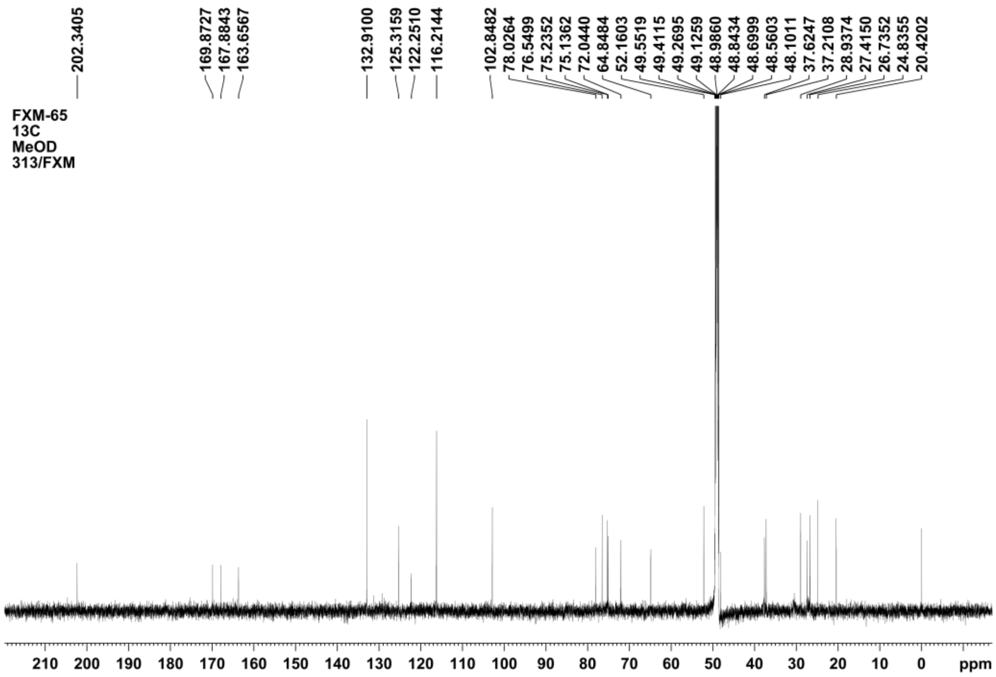

Figure S20. ^{13}C NMR spectrum of **3** (150 MHz, CD_3OD)

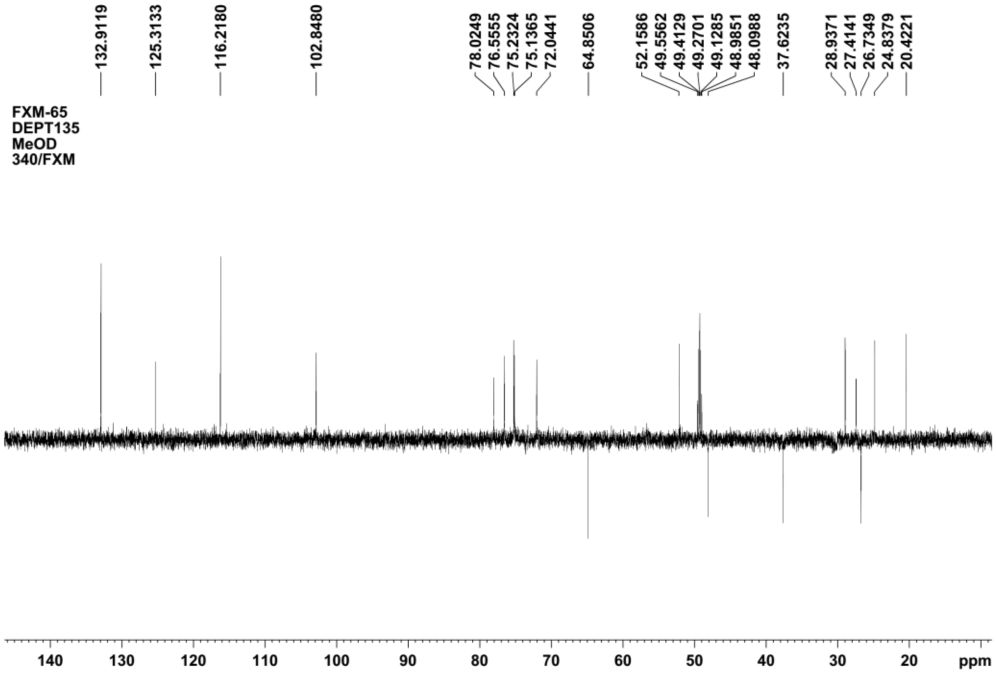

Figure S21. DEPT 135 spectrum of **3**

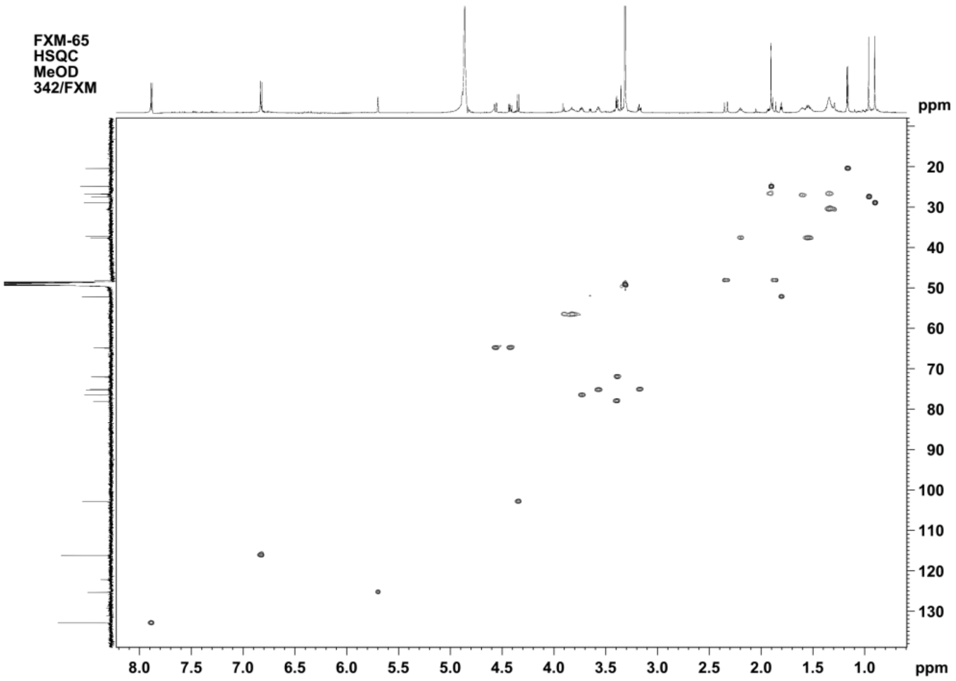

Figure S22. HSQC spectrum of **3**

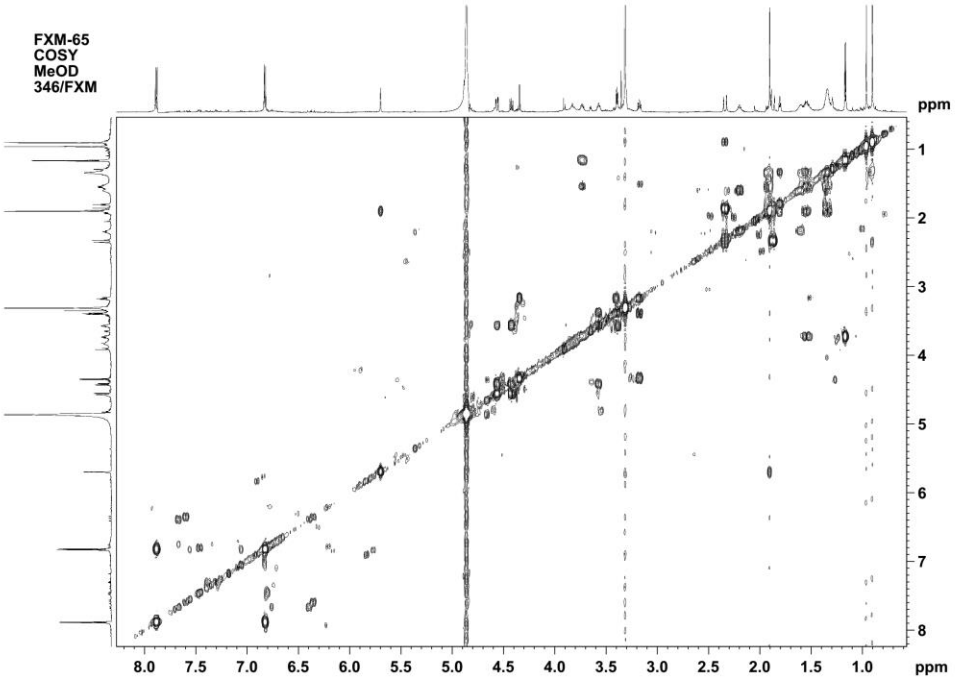

Figure S23. ^{1}H-^{1}H COSY spectrum of **3**

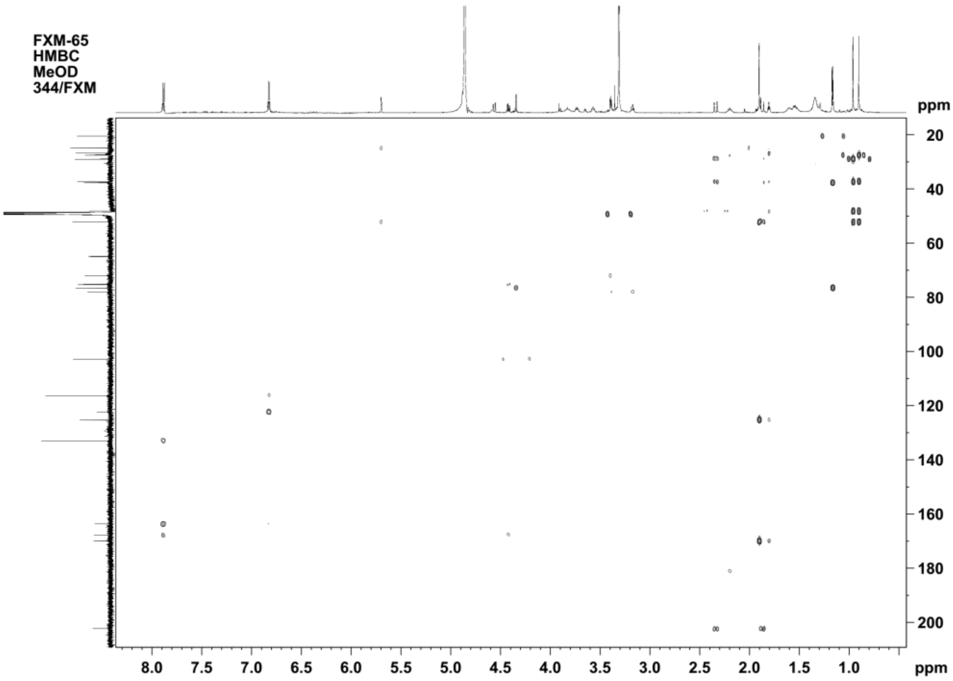

Figure S24. HMBC spectrum of **3**

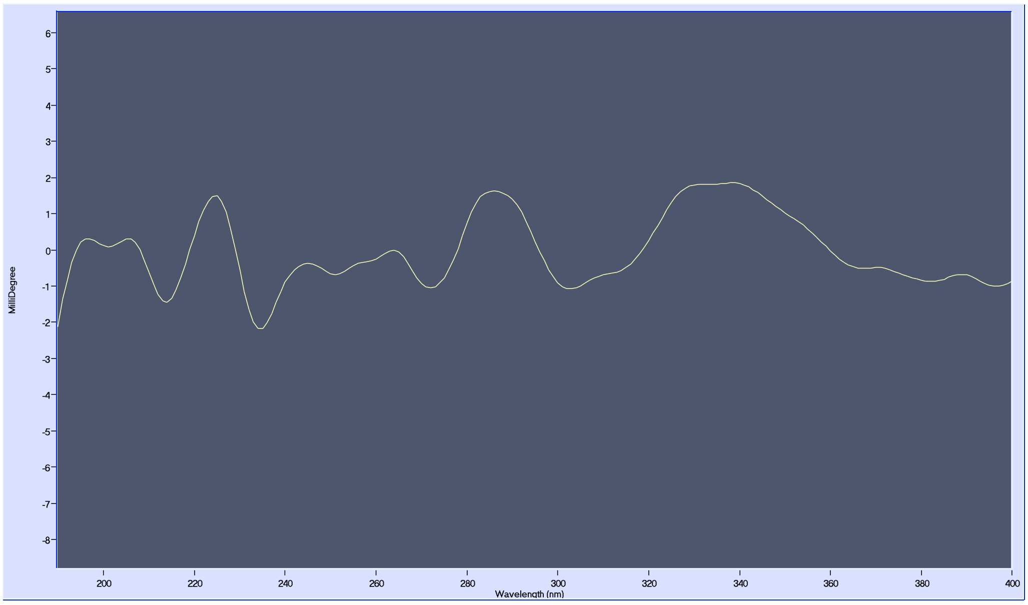

Figure S25. CD spectrum of **3**

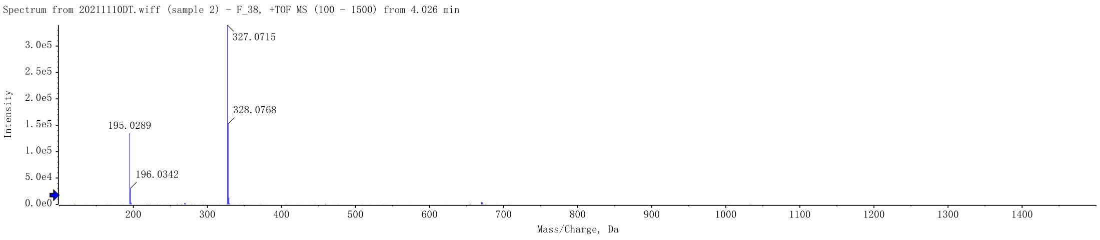

Figure S26. HR-ESI-MS spectrum of **12**

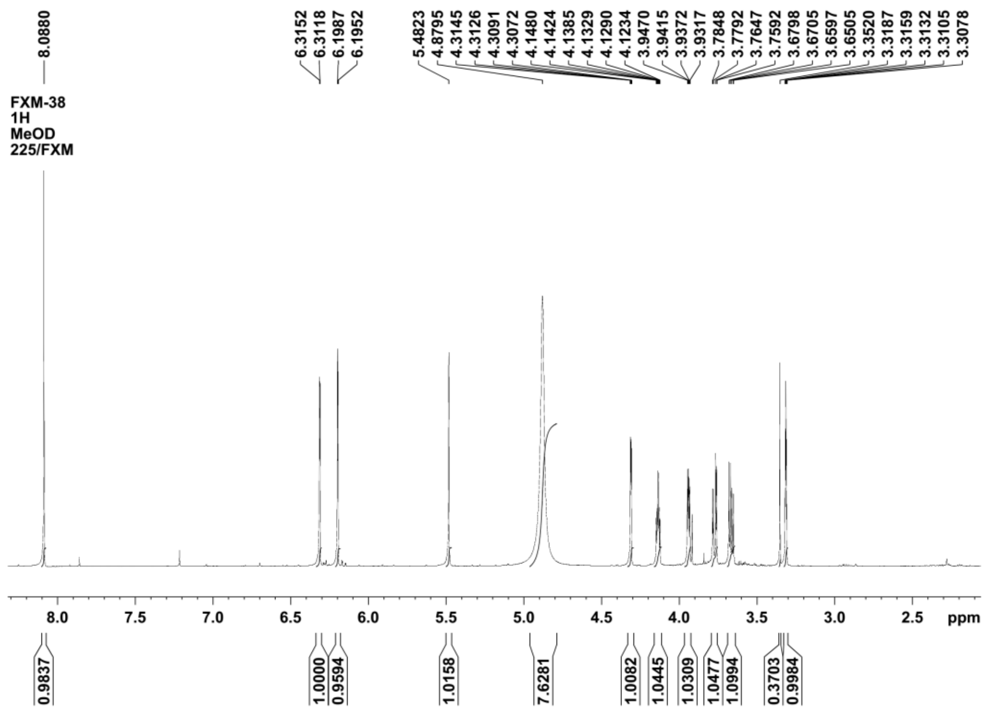

Figure S27. ^{1}H NMR spectrum of **12** (600 MHz, CD_3OD)

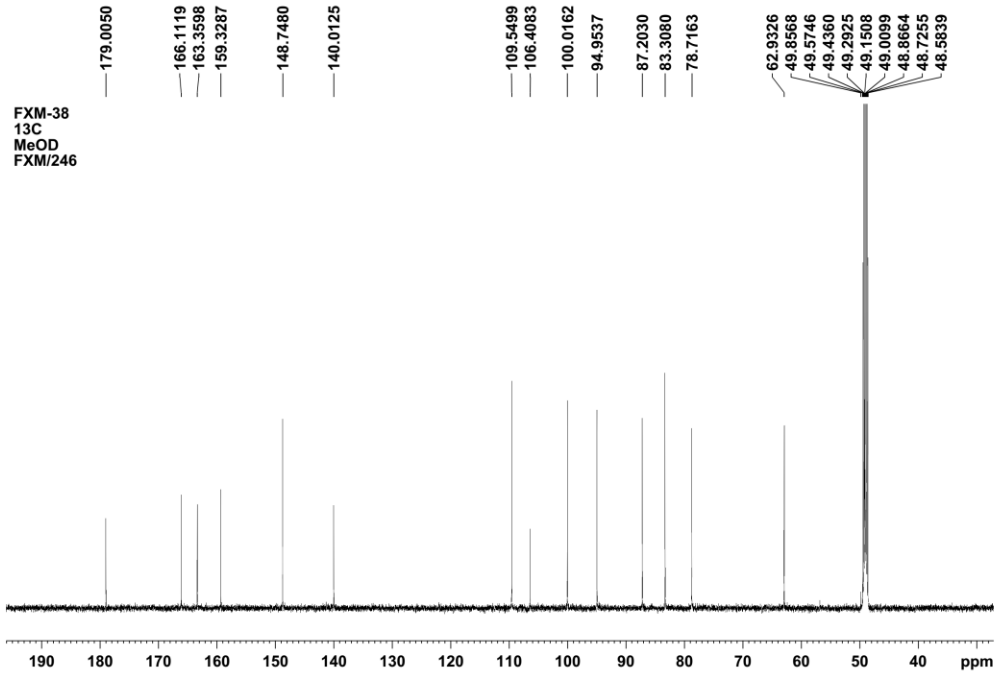

Figure S28. ^{13}C NMR spectrum of **12** (150 MHz, CD_3OD)

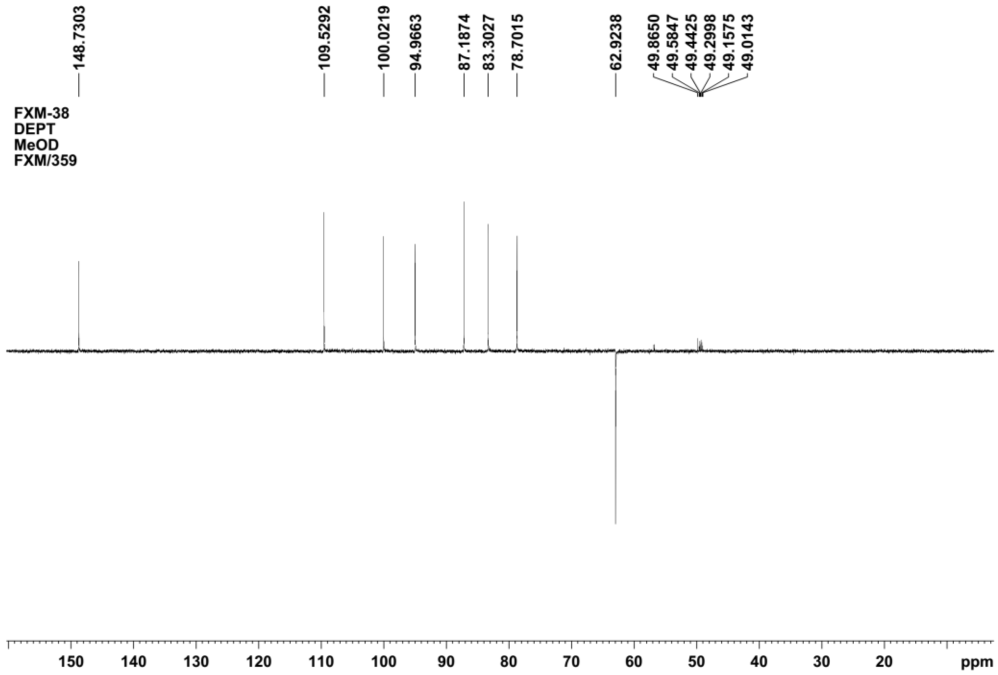

Figure S29. DEPT 135 spectrum of **12**

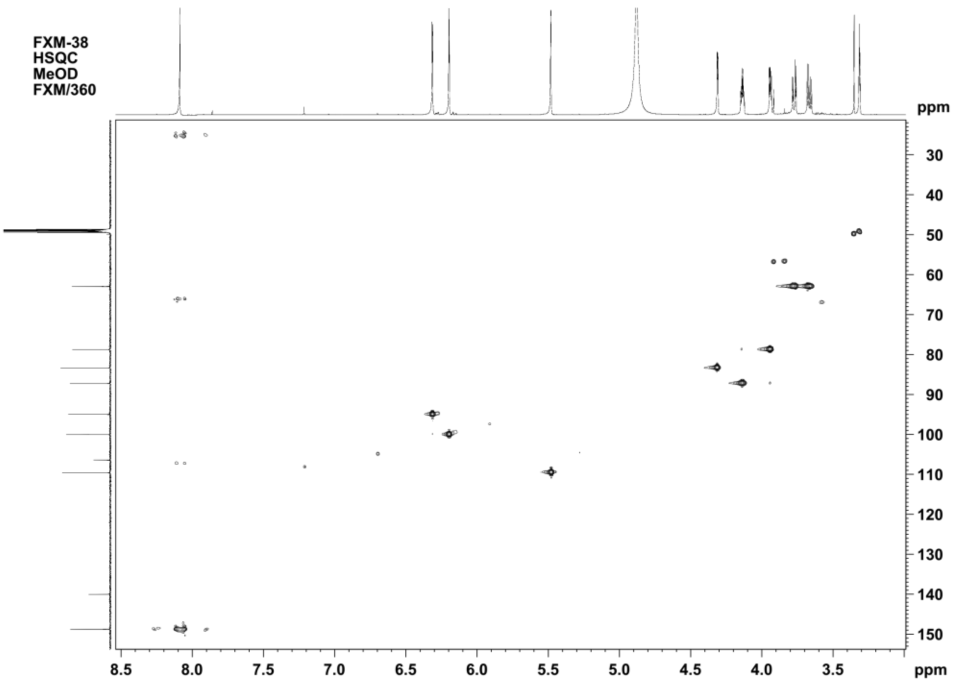

Figure S30. HSQC spectrum of **12**

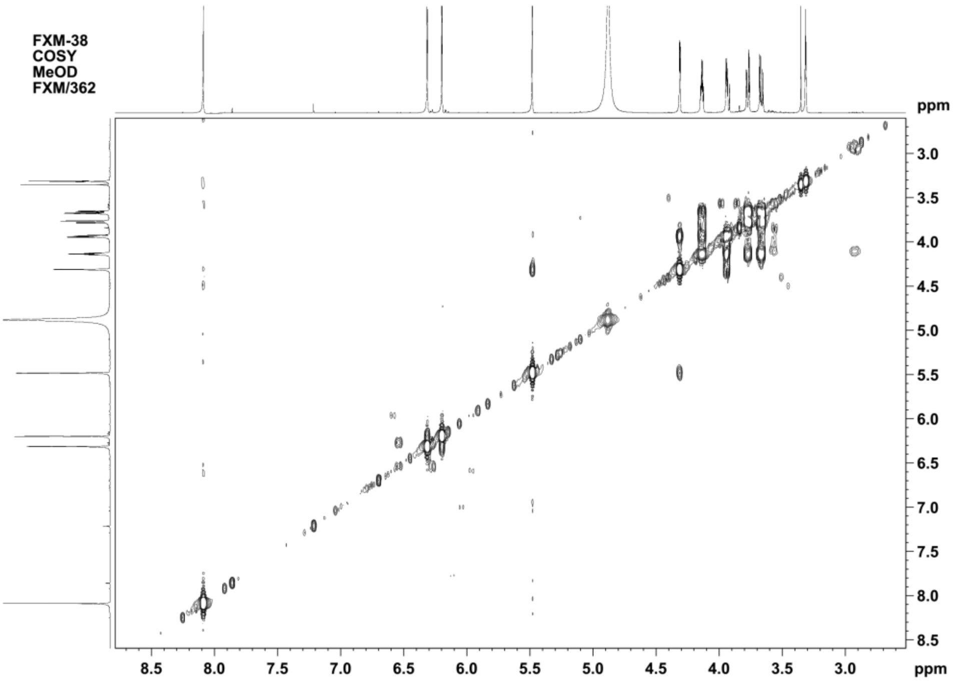

Figure S31. ^{1}H-^{1}H COSY spectrum of **12**

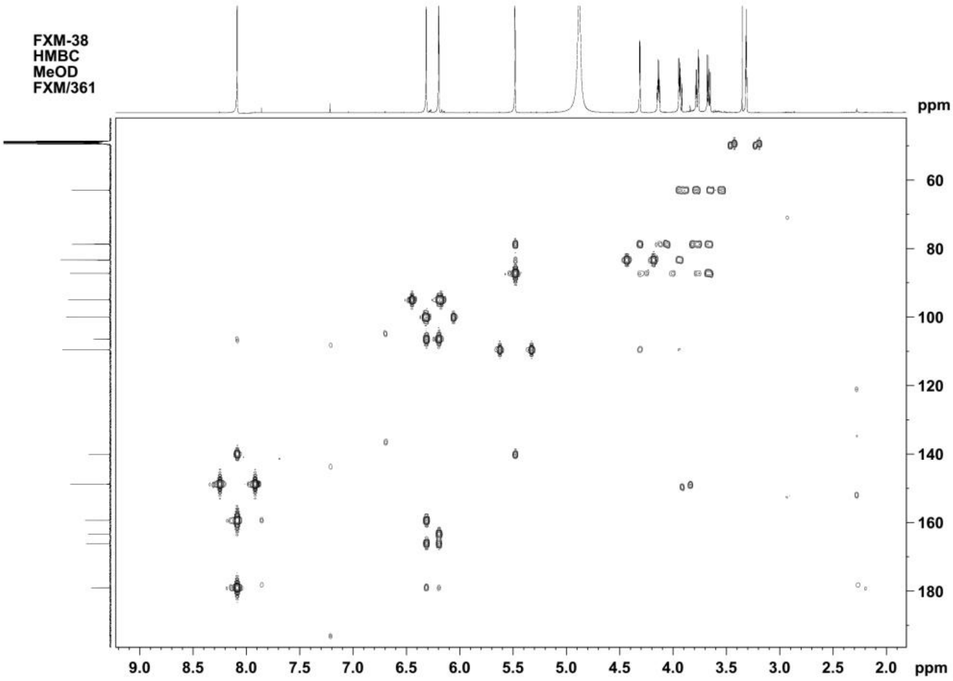

Figure S32. HMBC spectrum of **12**

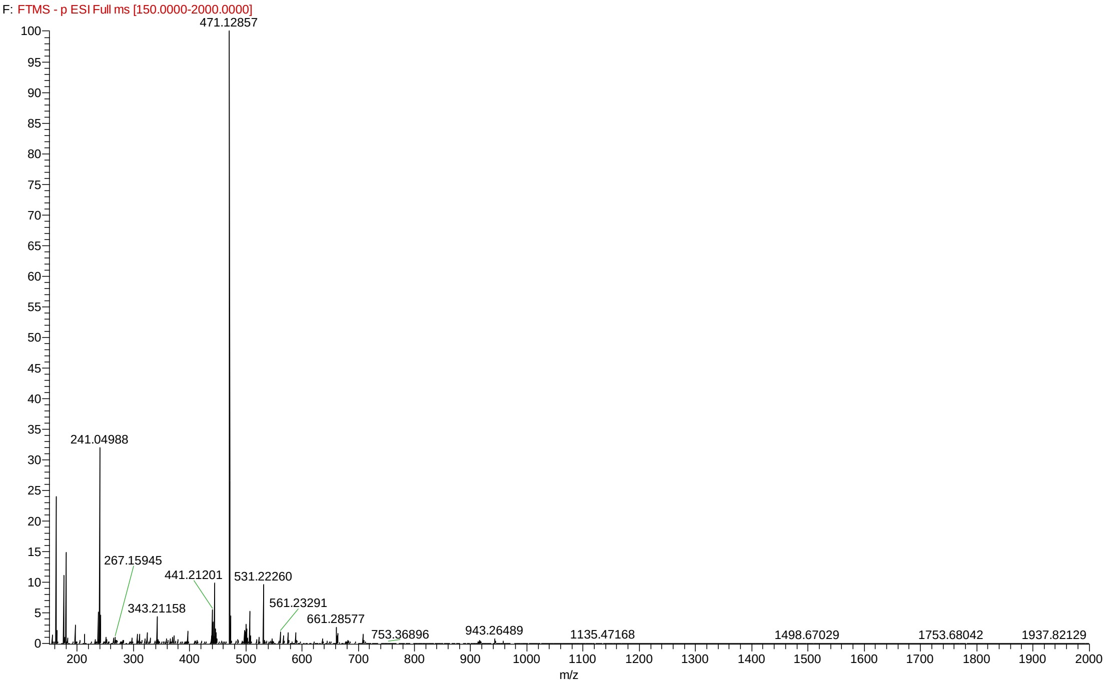

Figure S33. HR-ESI-MS spectrum of **29**

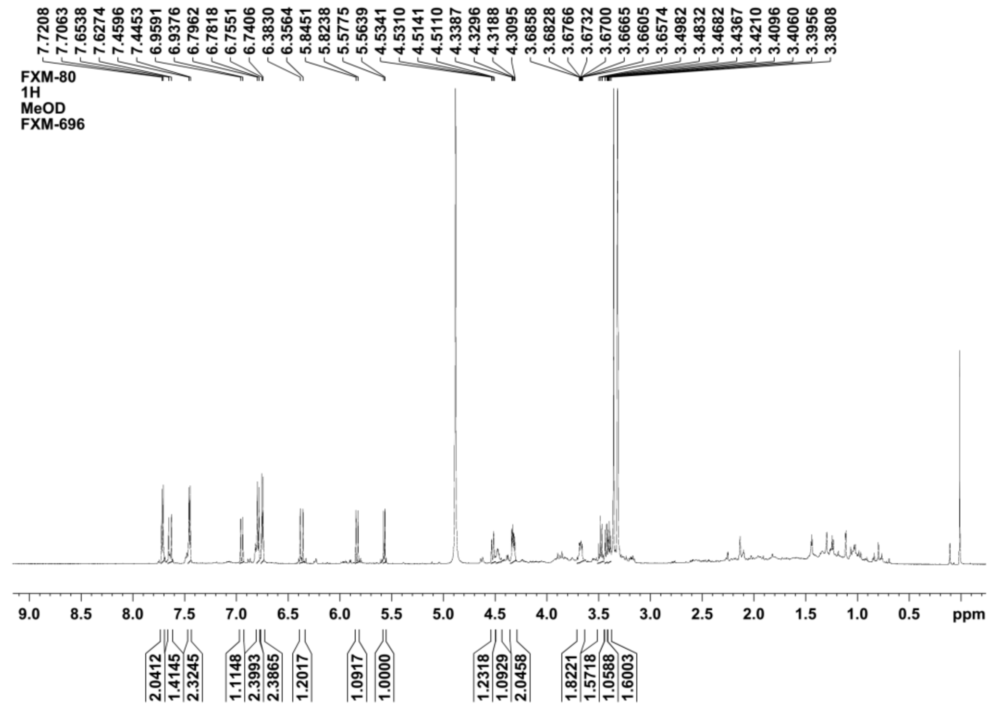

Figure S34. ^{1}H NMR spectrum of **29** (600 MHz, CD_3OD)

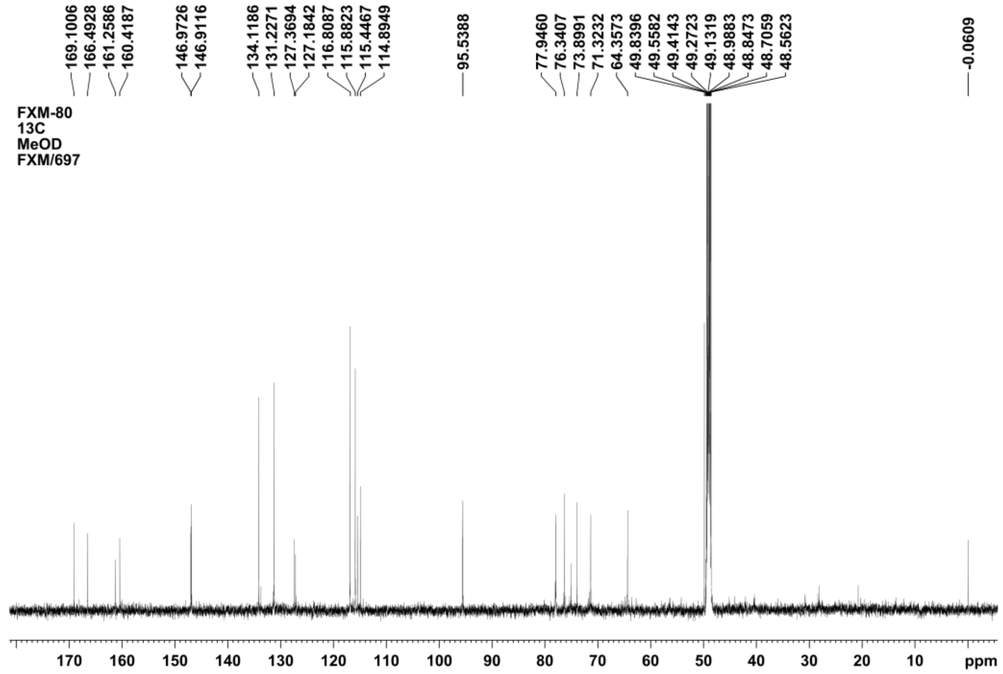

Figure S35. ^{13}C NMR spectrum of **29** (150 MHz, CD_3OD)

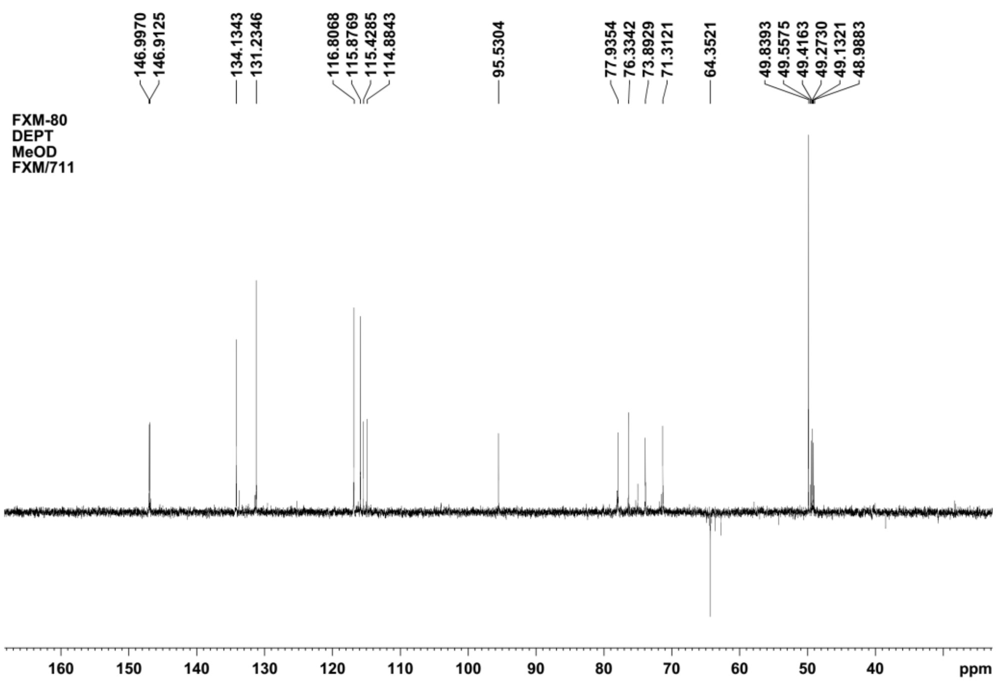

Figure S36. DEPT 135 spectrum of **29**

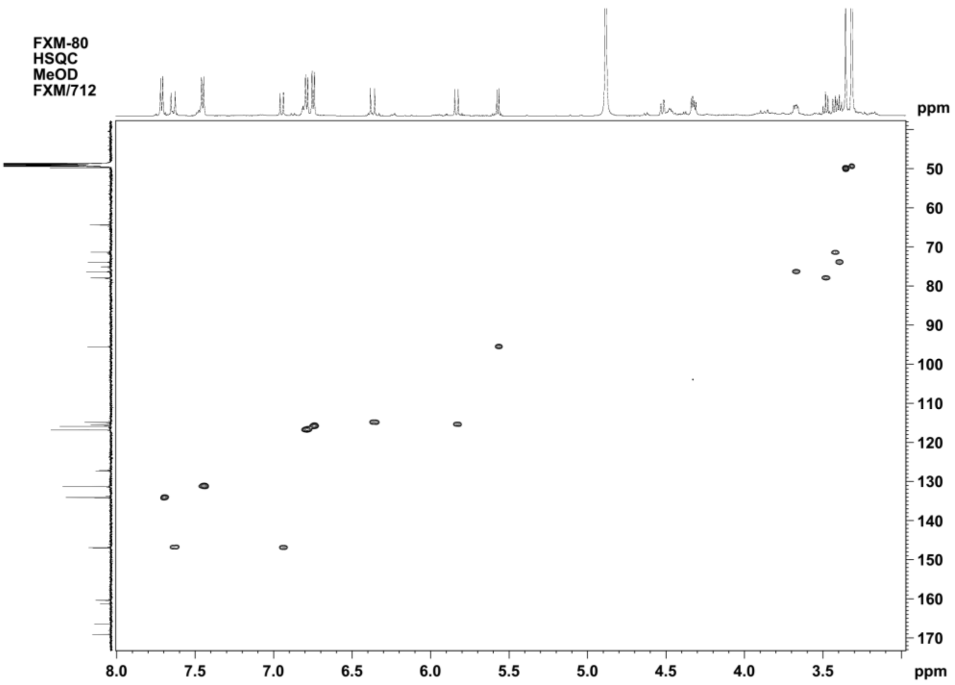

Figure S37. HSQC spectrum of **29**

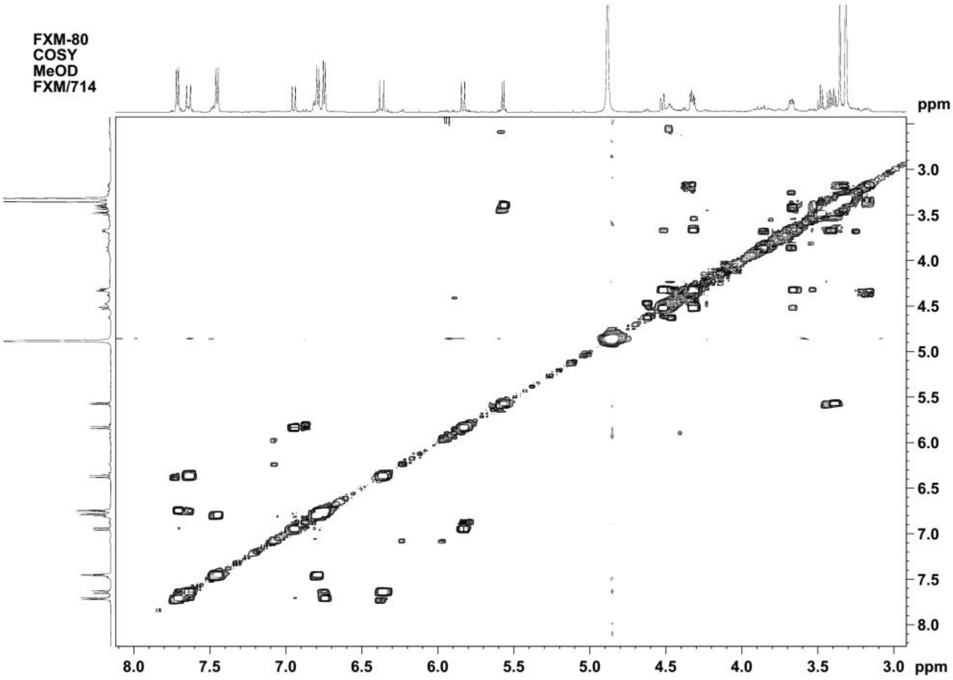

Figure S38. ^{1}H-^{1}H COSY spectrum of **29**

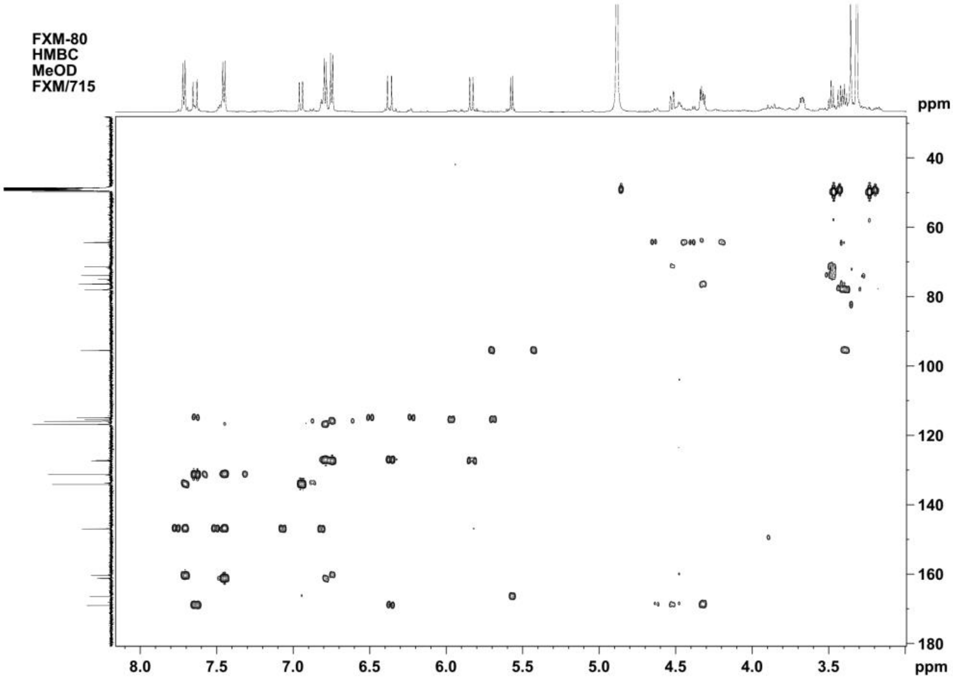

Figure S39. HMBC spectrum of **29**

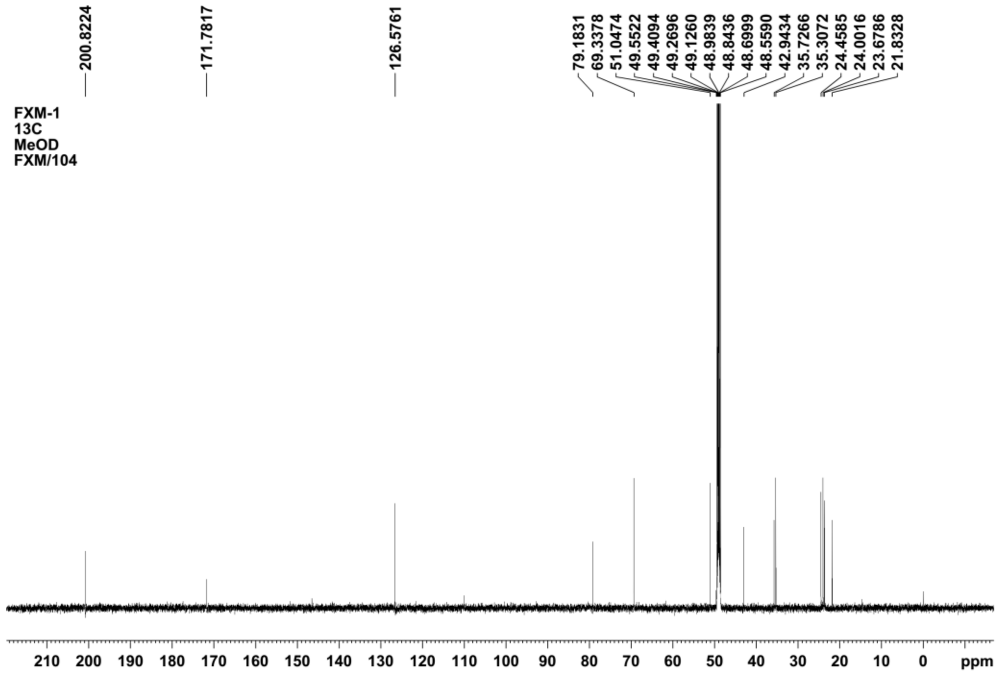

Figure S40. ^{13}C NMR spectrum of **4** (150 MHz, CD_3OD)

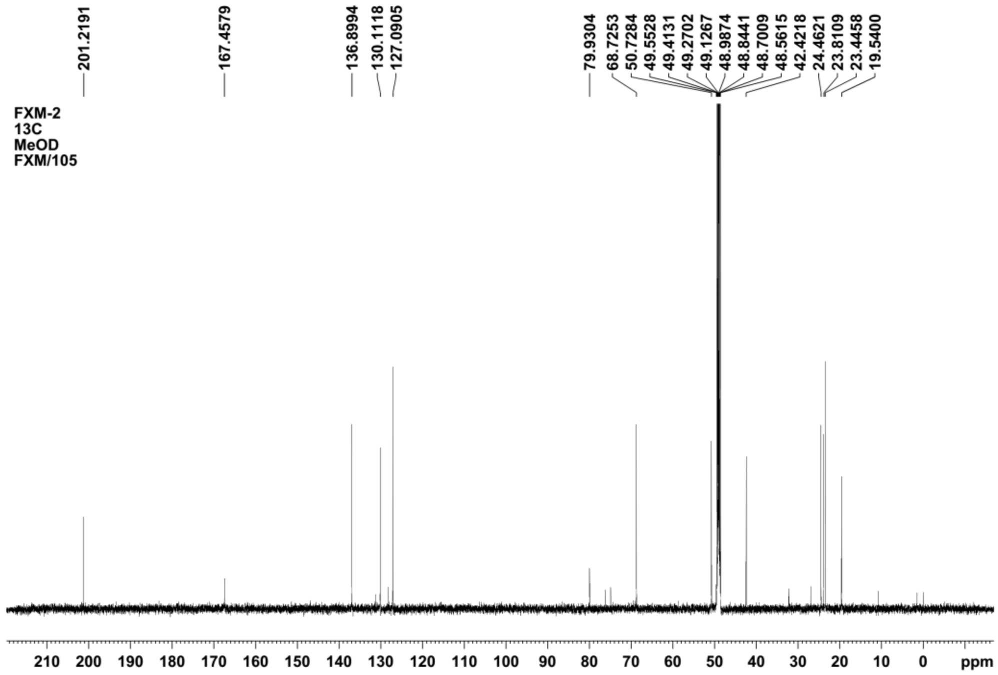

Figure S41. ^{13}C NMR spectrum of **5** (150 MHz, CD_3OD)

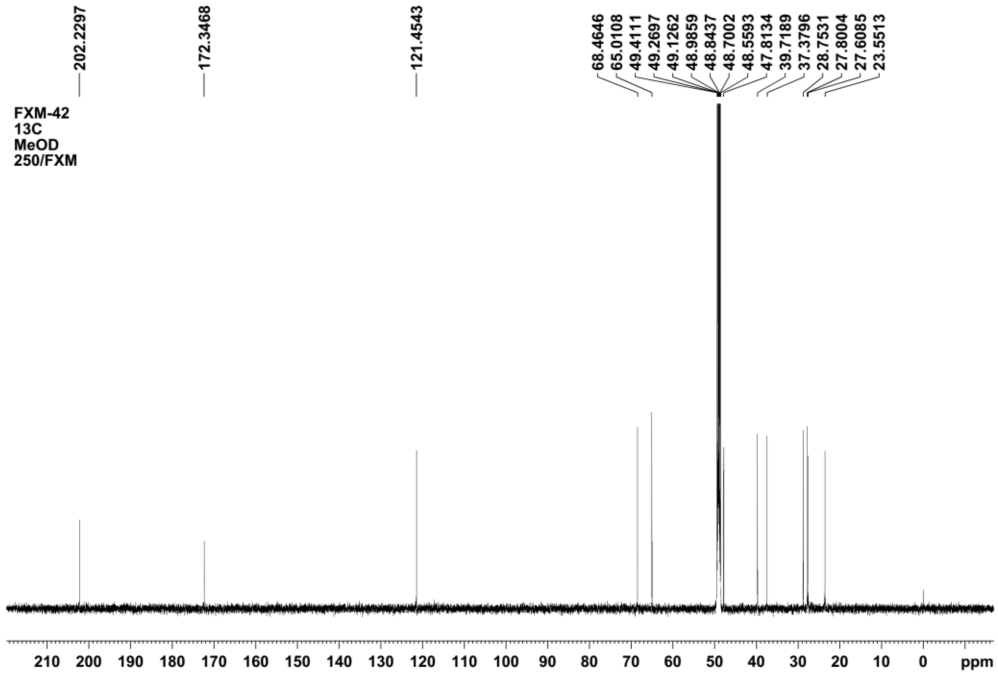

Figure S42. ^{13}C NMR spectrum of **6** (150 MHz, CD_3OD)

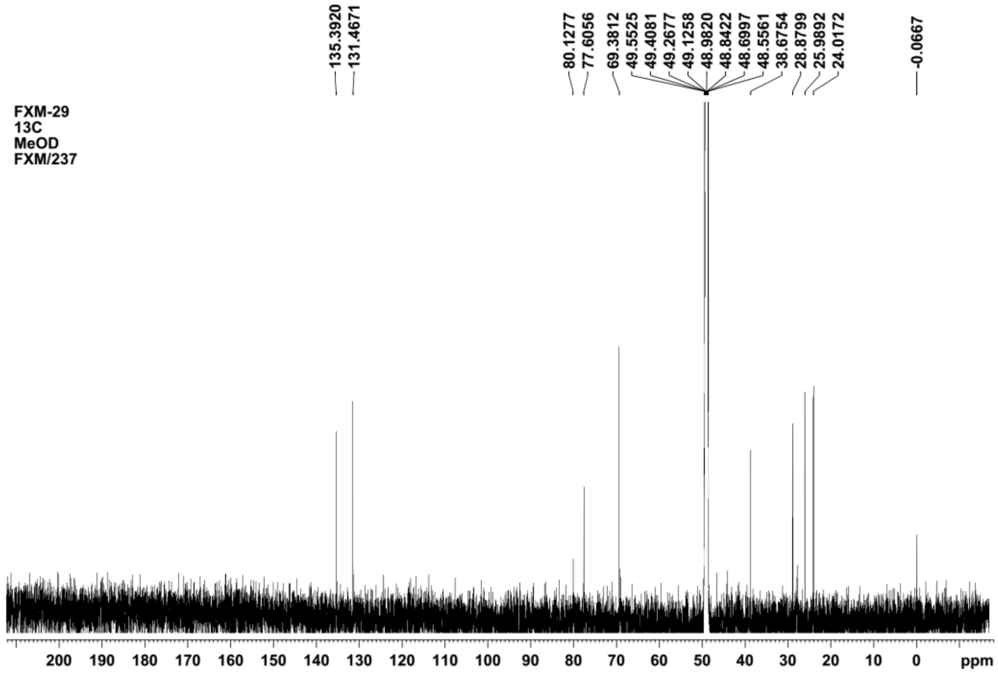

Figure S43. ^{13}C NMR spectrum of **7** (150 MHz, CD_3OD)

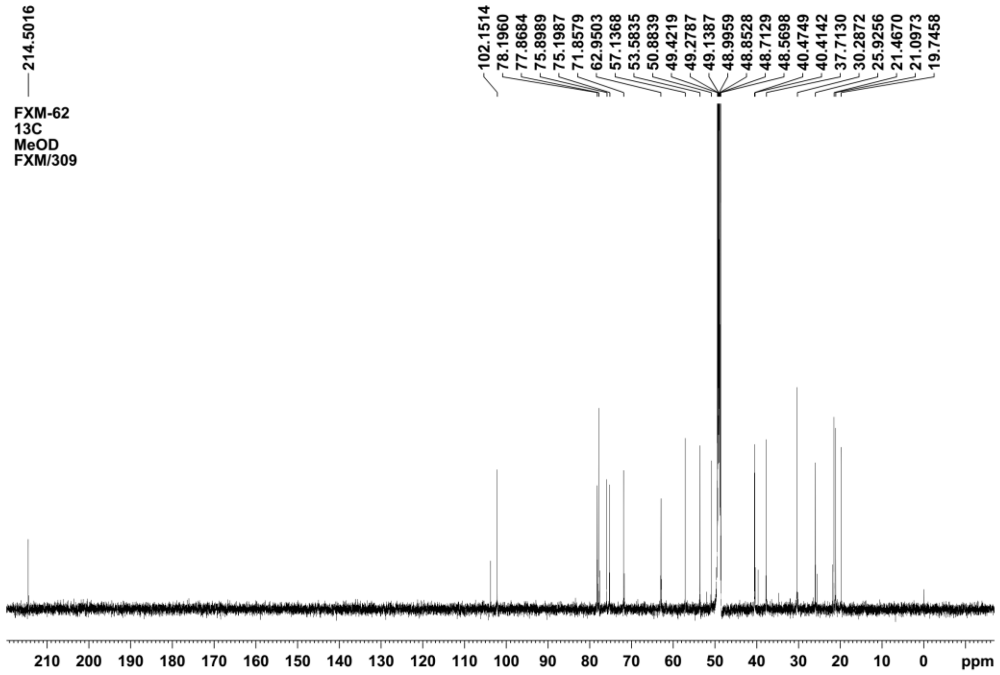

Figure S44. ^{13}C NMR spectrum of **8** (150 MHz, CD_3OD)

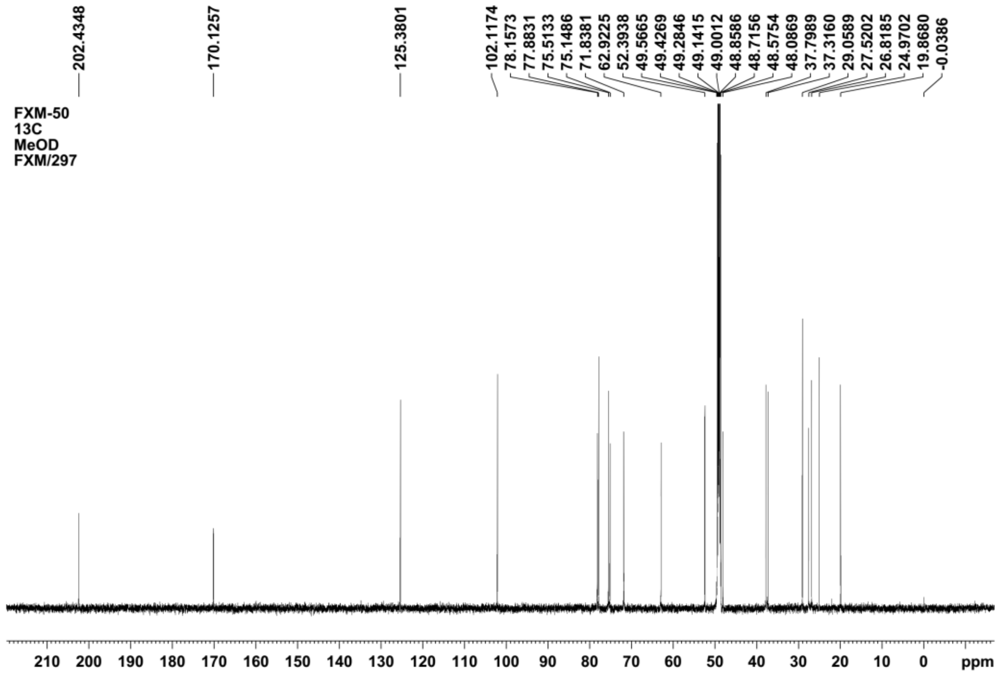

Figure S45. ^{13}C NMR spectrum of **9** (150 MHz, CD_3OD)

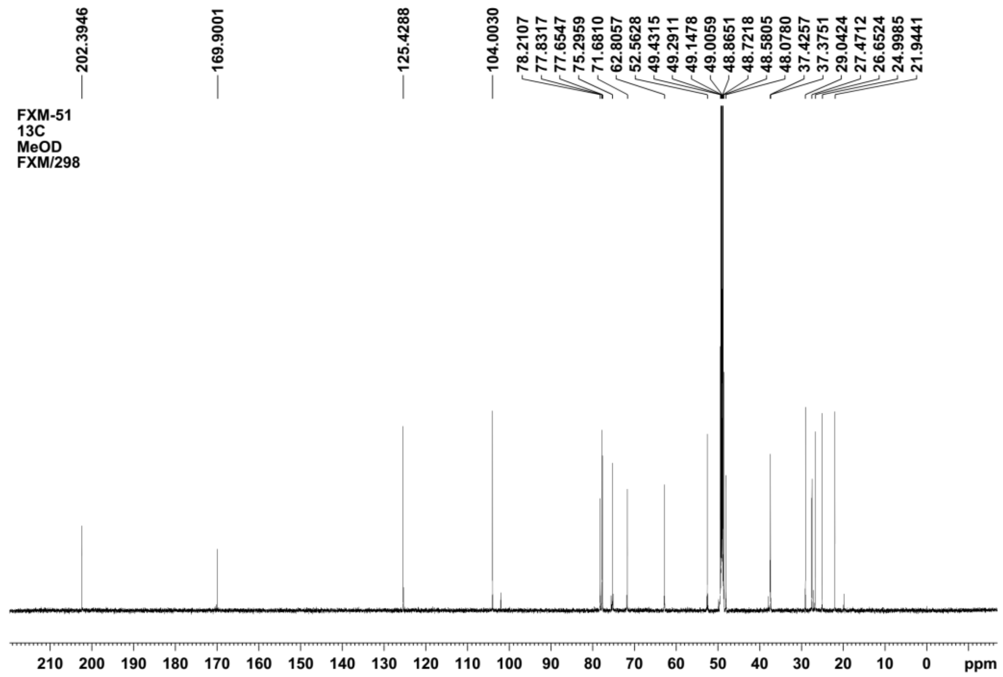

Figure S46. ^{13}C NMR spectrum of **10** (150 MHz, CD_3OD)

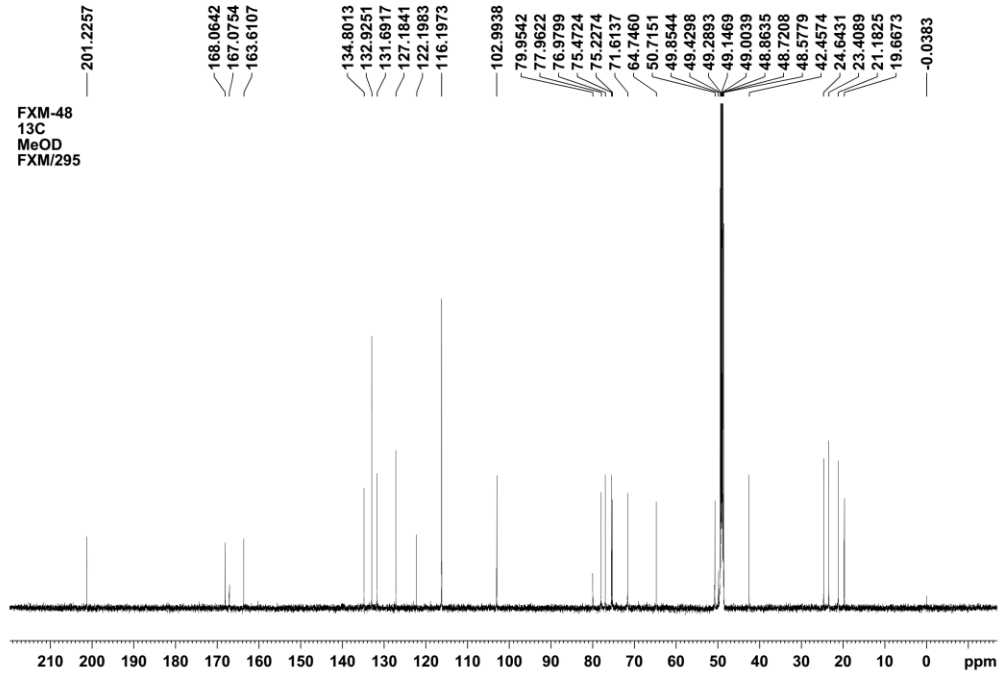

Figure S47. ^{13}C NMR spectrum of **11** (150 MHz, CD_3OD)

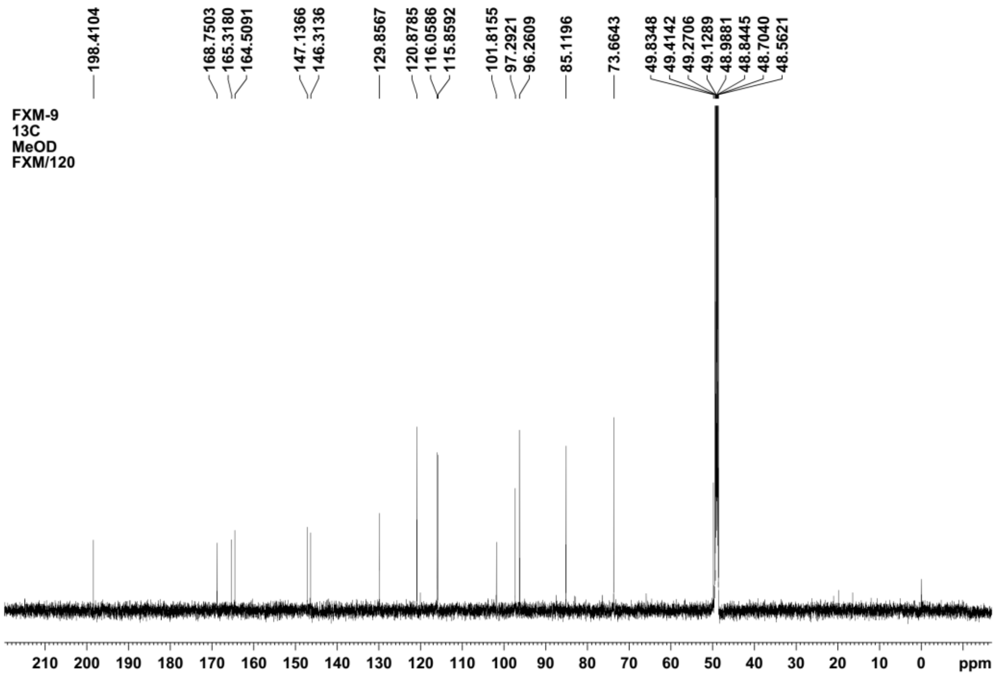

Figure S48. ^{13}C NMR spectrum of **13** (150 MHz, CD_3OD)

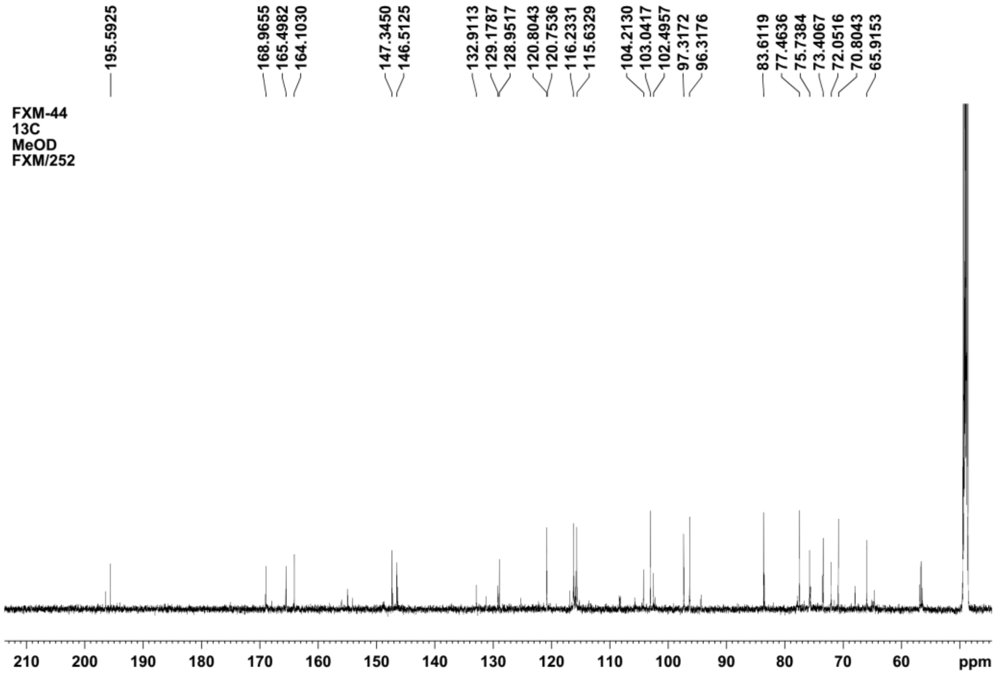

Figure S49. ^{13}C NMR spectrum of **14** (150 MHz, CD_3OD)

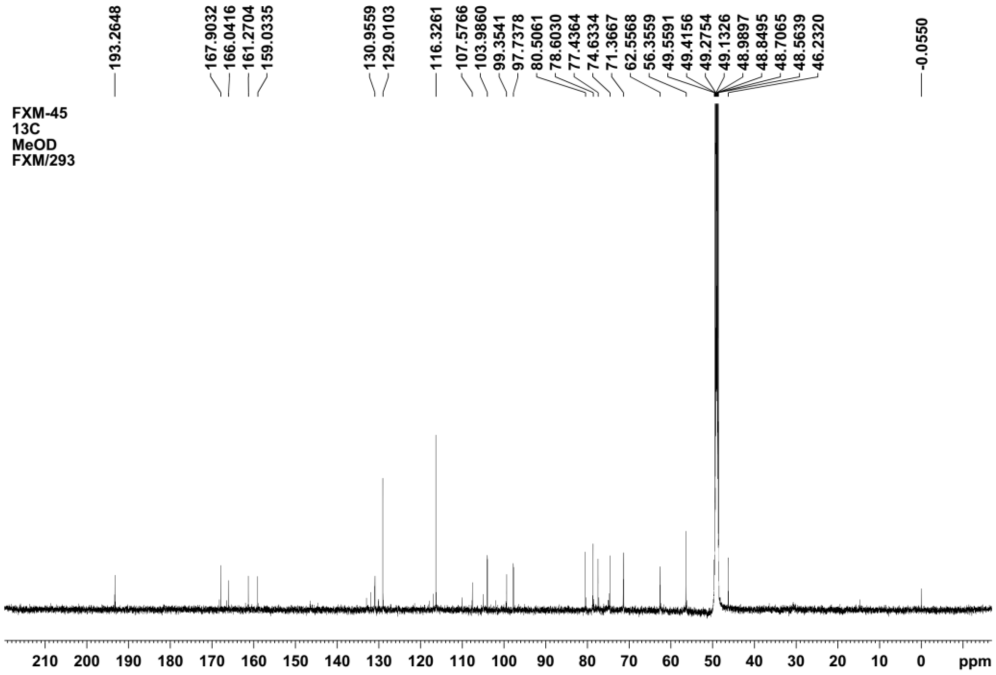

Figure S50. ^{13}C NMR spectrum of **15** (150 MHz, CD_3OD)

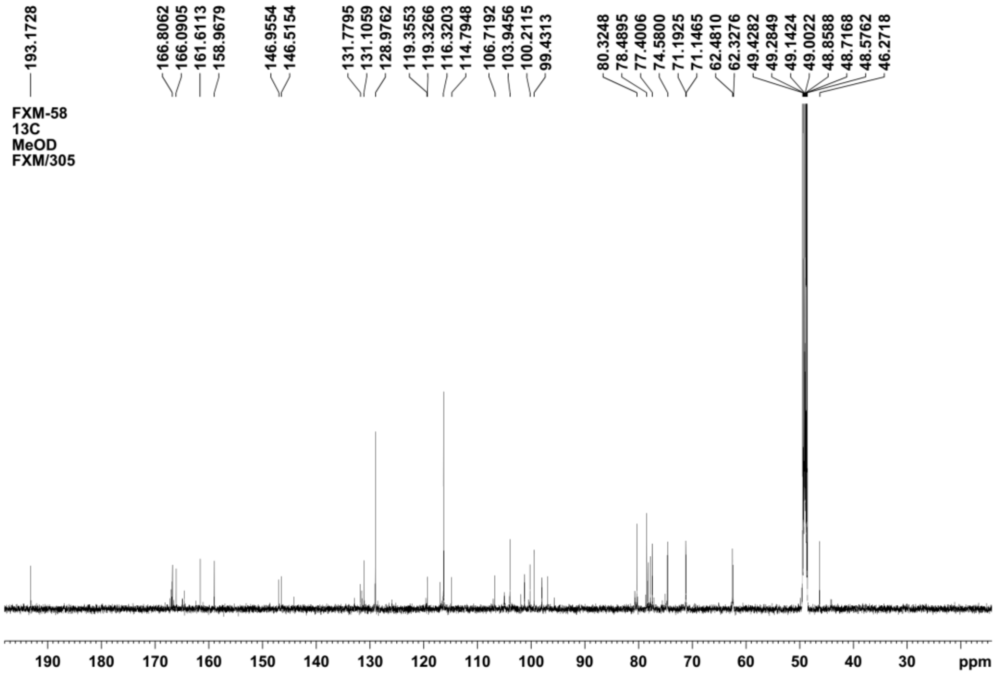

Figure S51. ^{13}C NMR spectrum of **16** (150 MHz, CD_3OD)

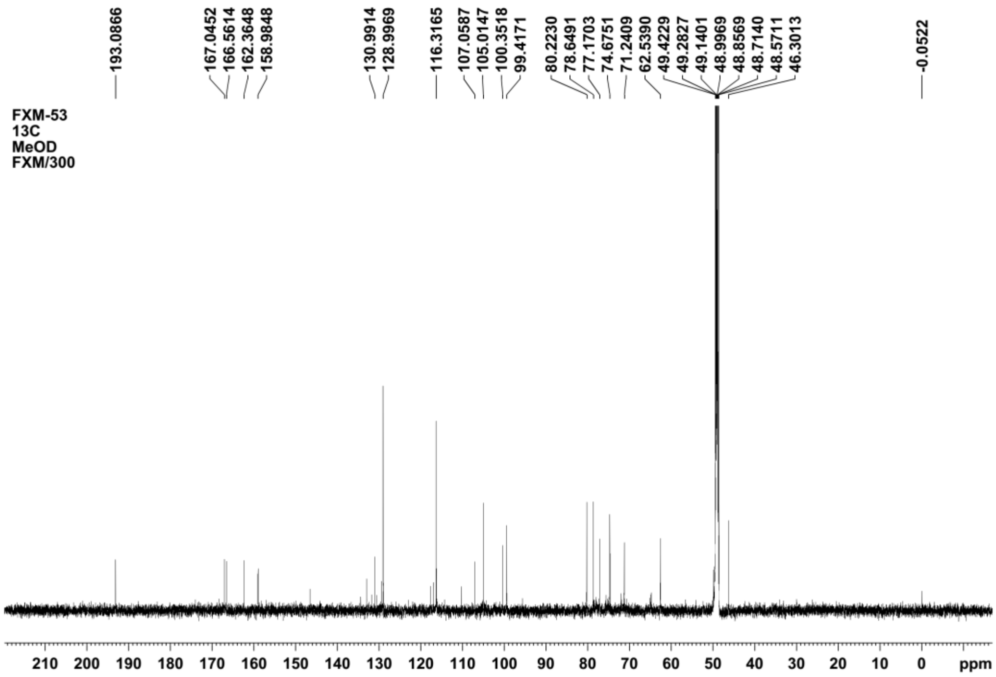

Figure S52. ^{13}C NMR spectrum of **17** (150 MHz, CD_3OD)

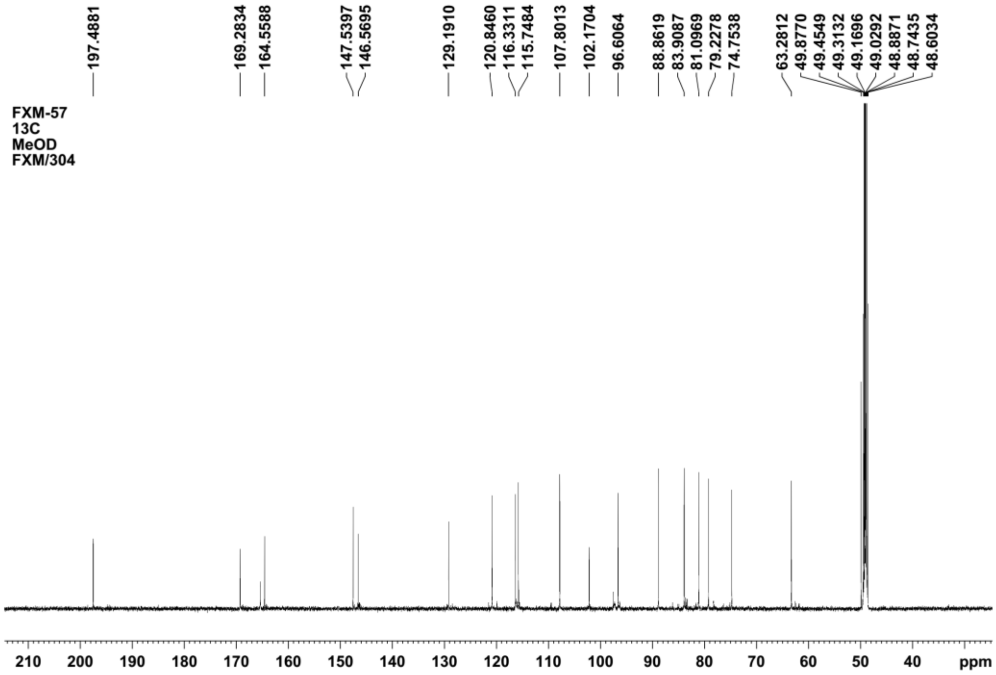

Figure S53. ^{13}C NMR spectrum of **18** (150 MHz, CD_3OD)

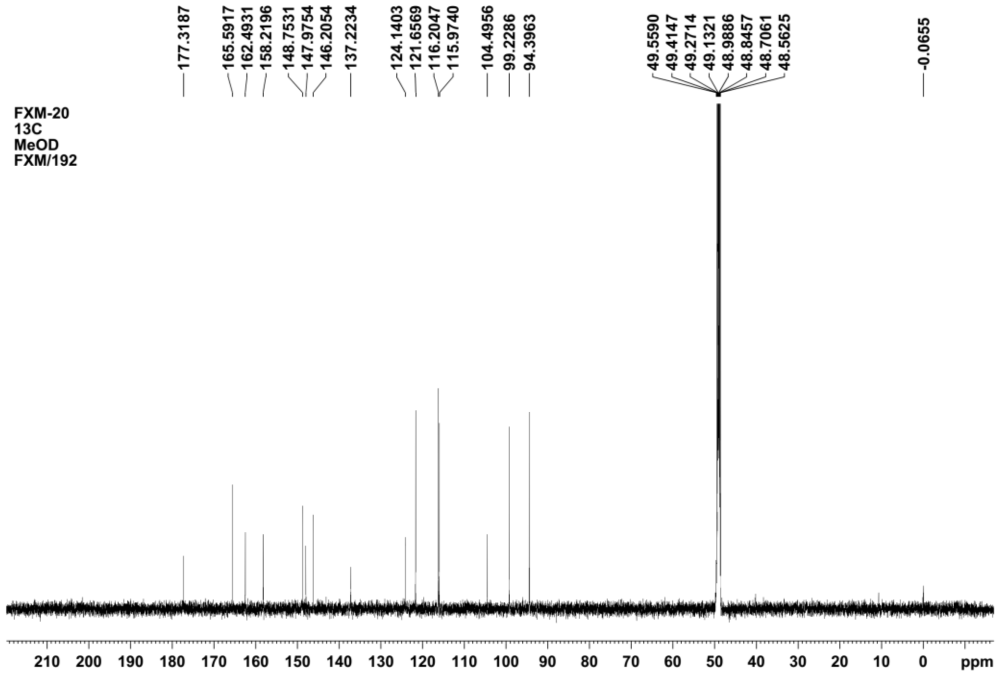

Figure S54. ^{13}C NMR spectrum of **19** (150 MHz, CD_3OD)

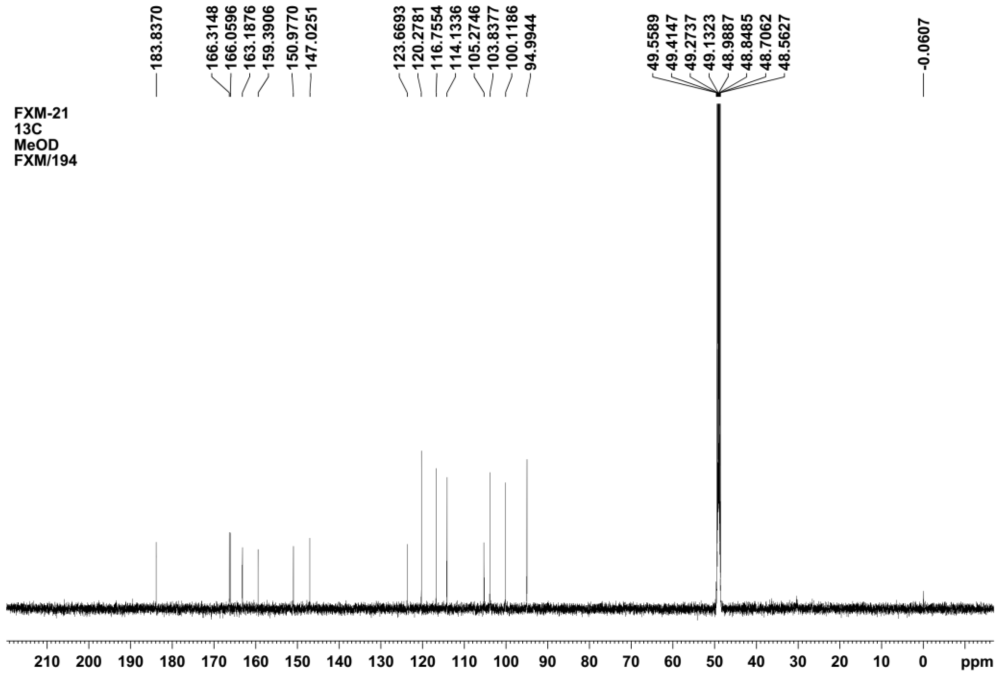

Figure S55. ^{13}C NMR spectrum of **20** (150 MHz, CD_3OD)

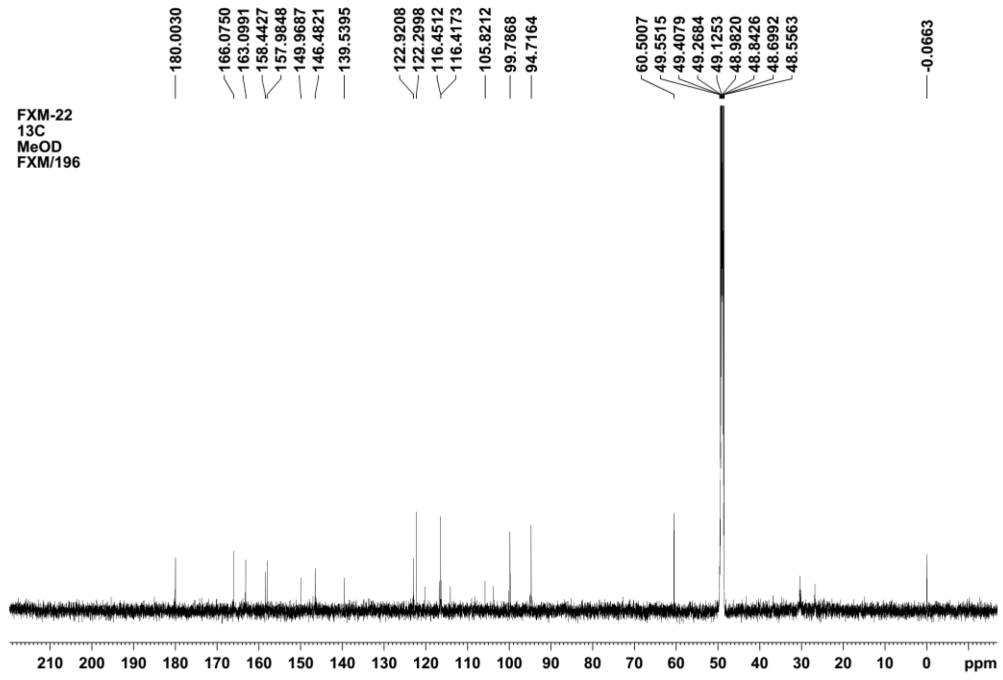

Figure S56. ^{13}C NMR spectrum of **21** (150 MHz, CD_3OD)

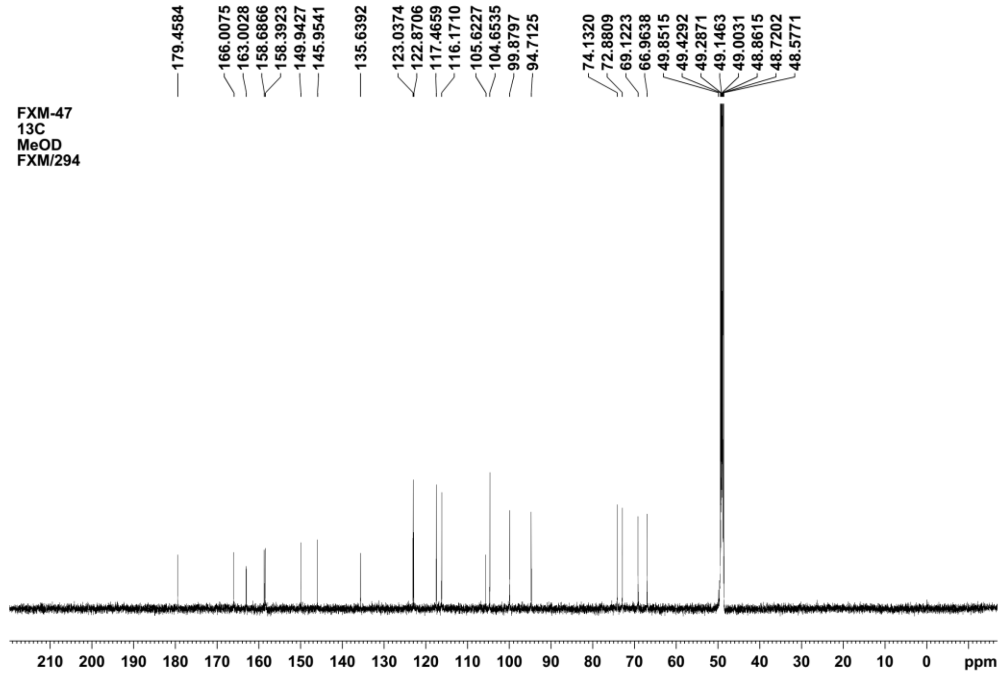

Figure S57. ^{13}C NMR spectrum of **22** (150 MHz, CD_3OD)

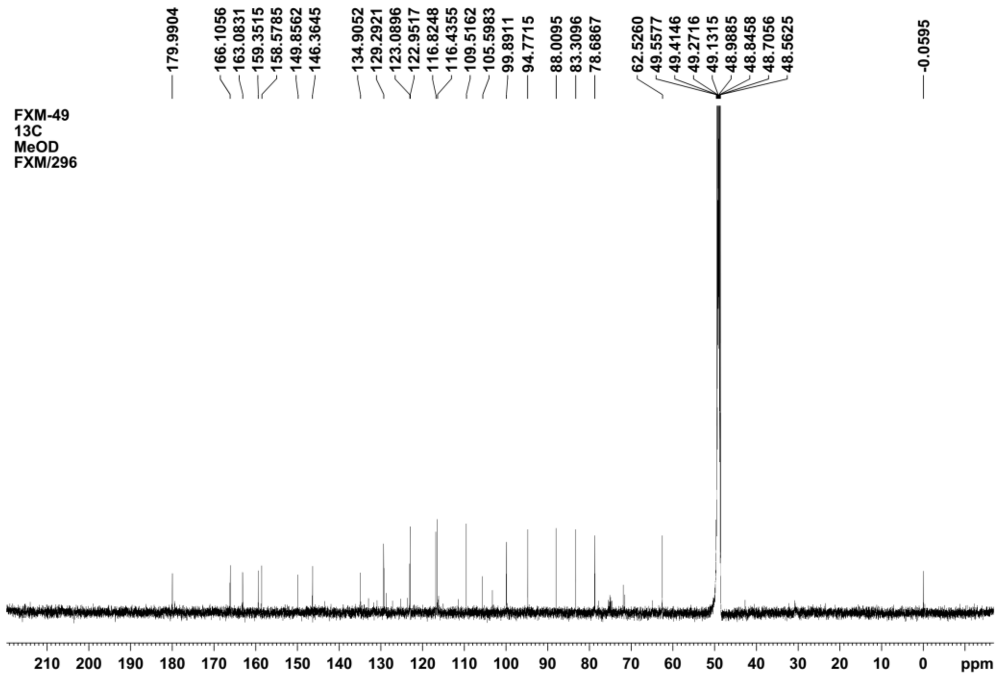

Figure S58. ^{13}C NMR spectrum of **23** (150 MHz, CD_3OD)

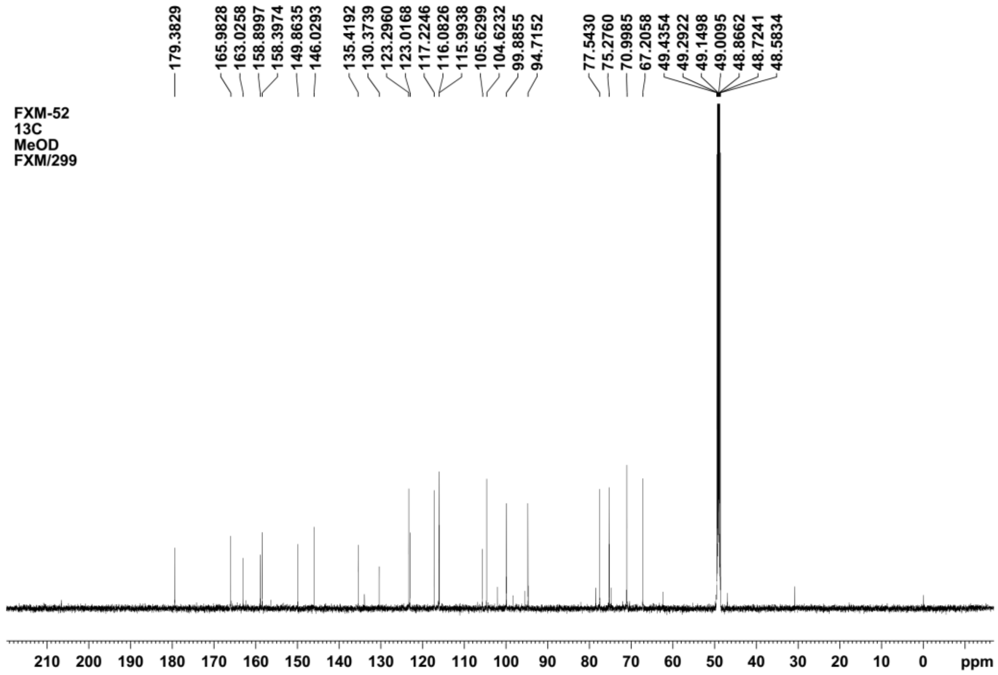

Figure S59. ^{13}C NMR spectrum of **24** (150 MHz, CD_3OD)

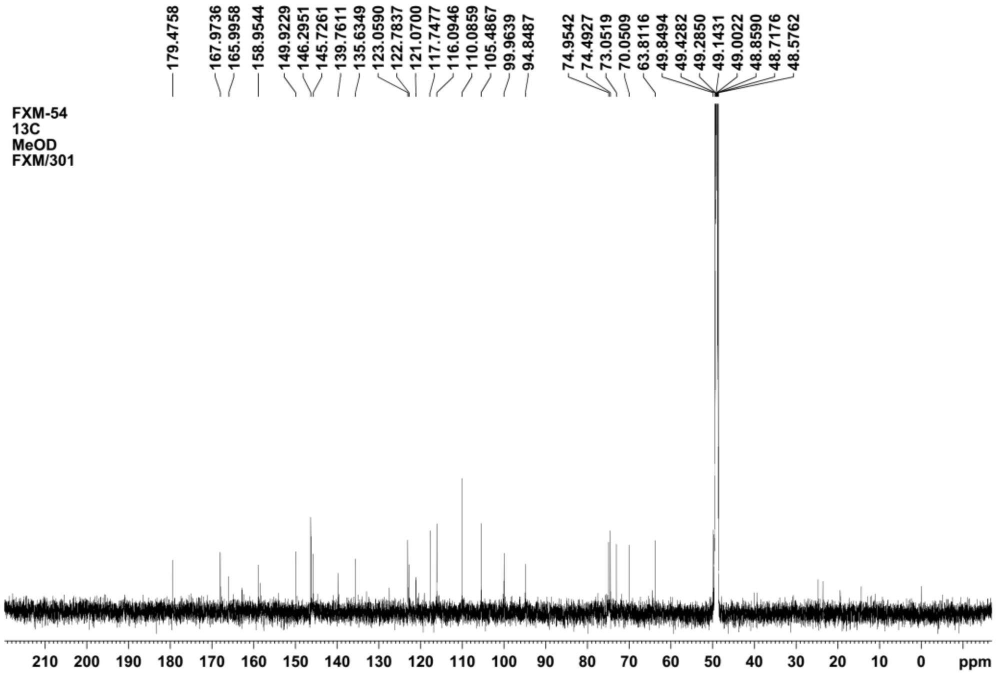

Figure S60. ^{13}C NMR spectrum of **25** (150 MHz, CD_3OD)

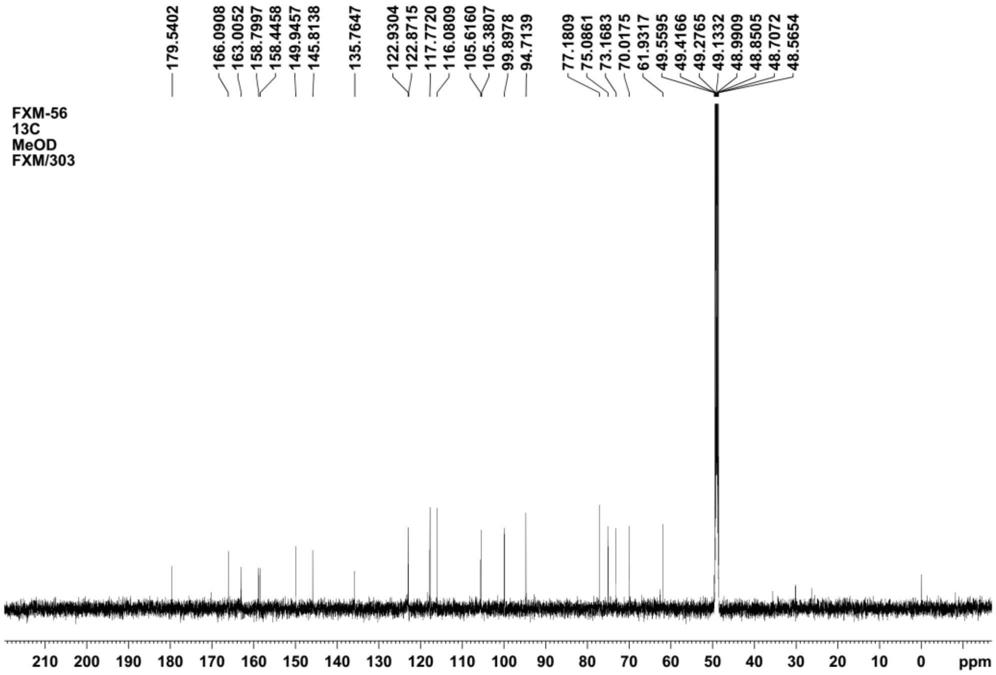

Figure S61. ^{13}C NMR spectrum of **26** (150 MHz, CD_3OD)

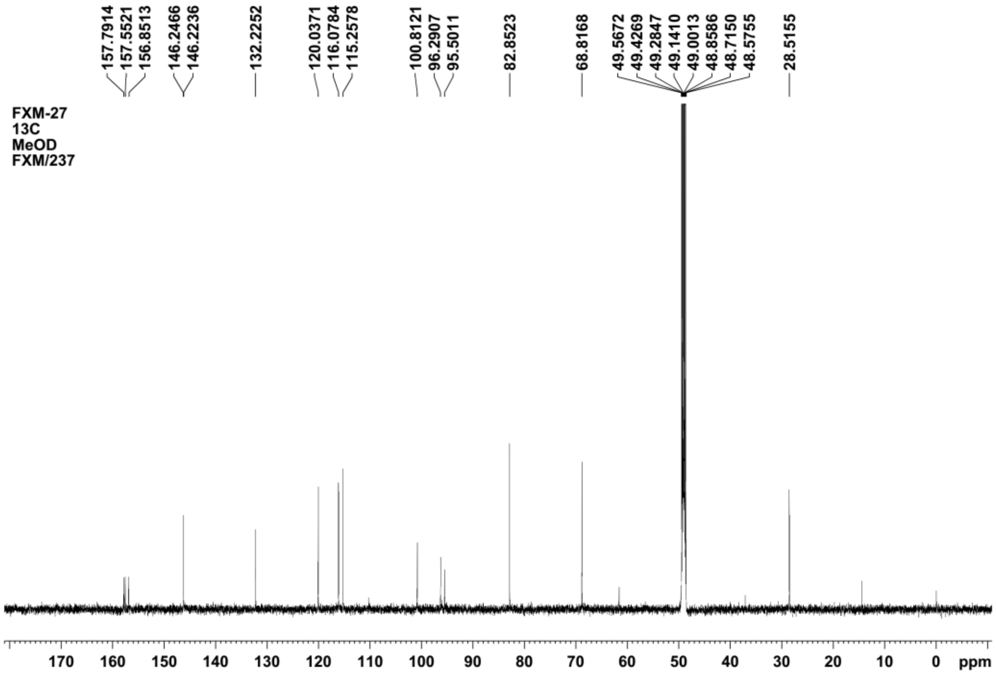

Figure S62. ^{13}C NMR spectrum of **27** (150 MHz, CD_3OD)

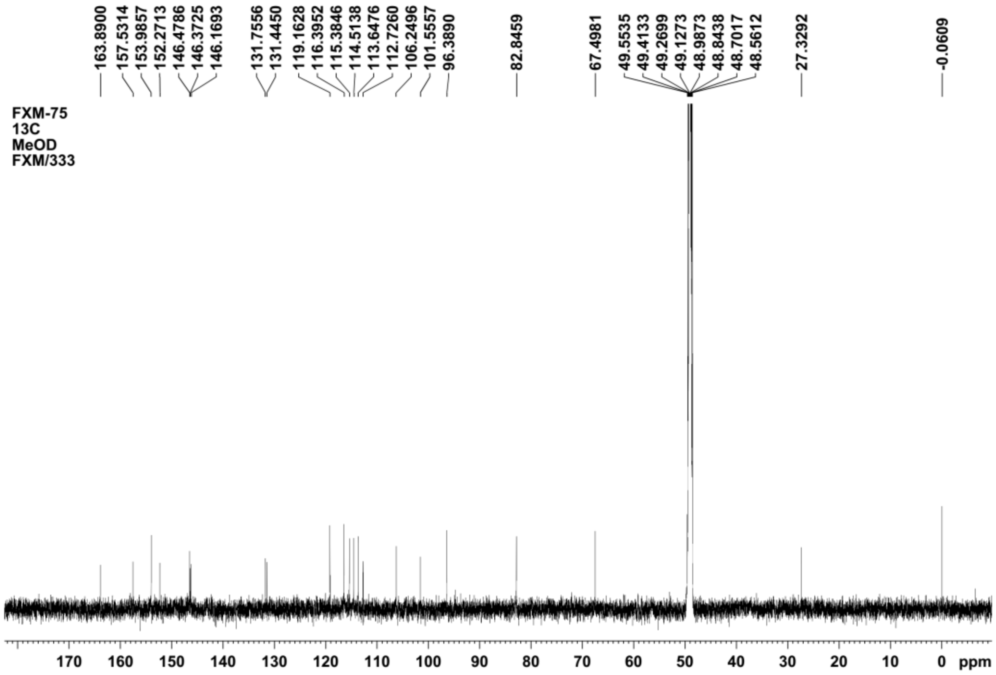

Figure S63. ^{13}C NMR spectrum of **28** (150 MHz, CD_3OD)

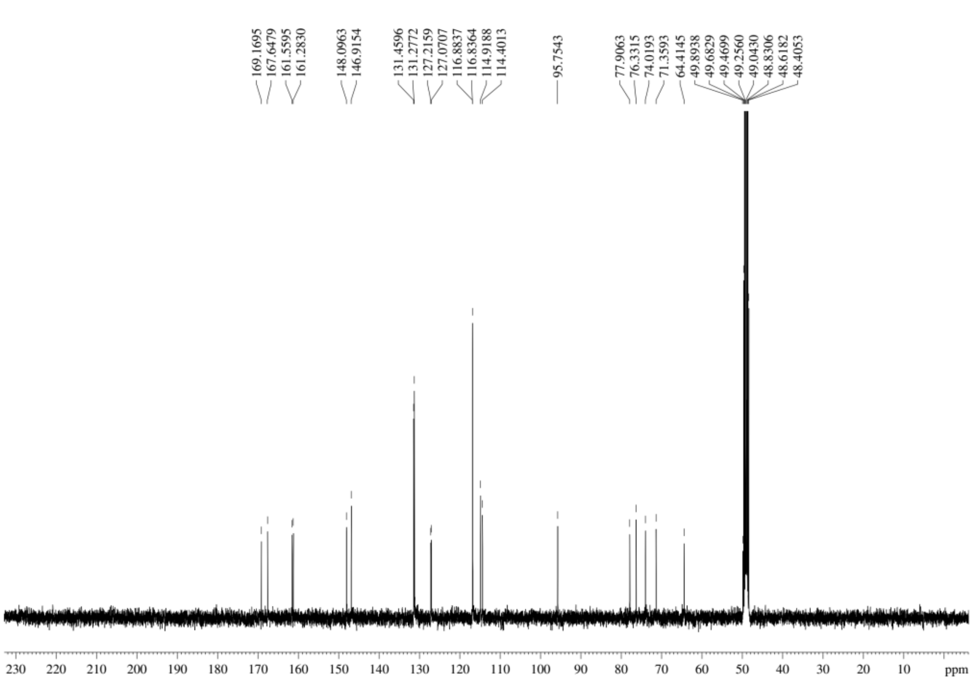

Figure S64. ^{13}C NMR spectrum of **30** (100 MHz, CD_3OD)

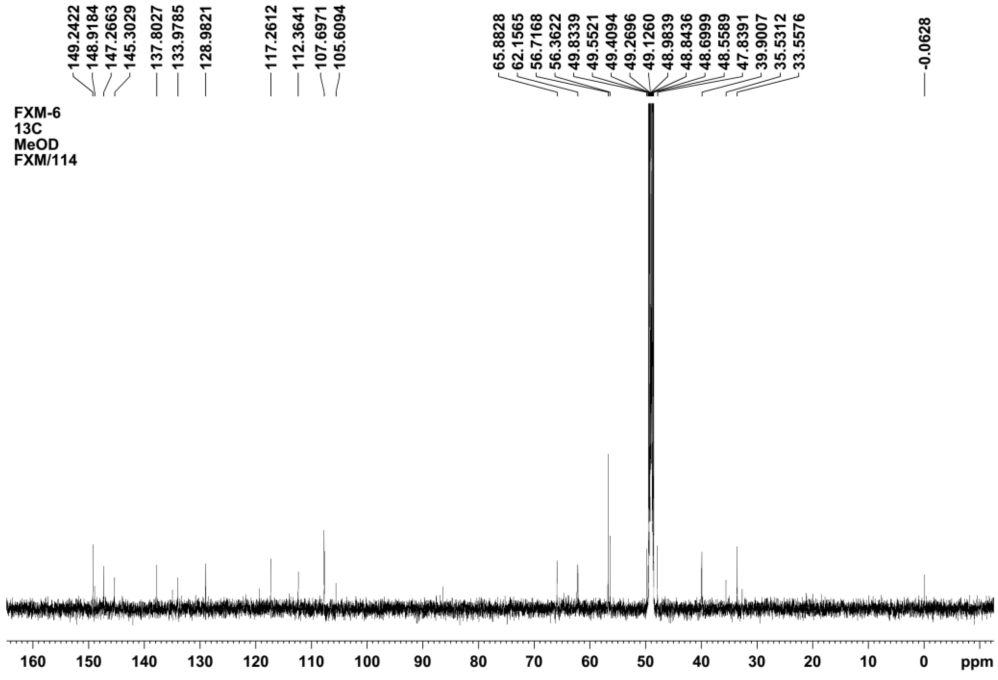

Figure S65. ^{13}C NMR spectrum of **31** (150 MHz, CD_3OD)

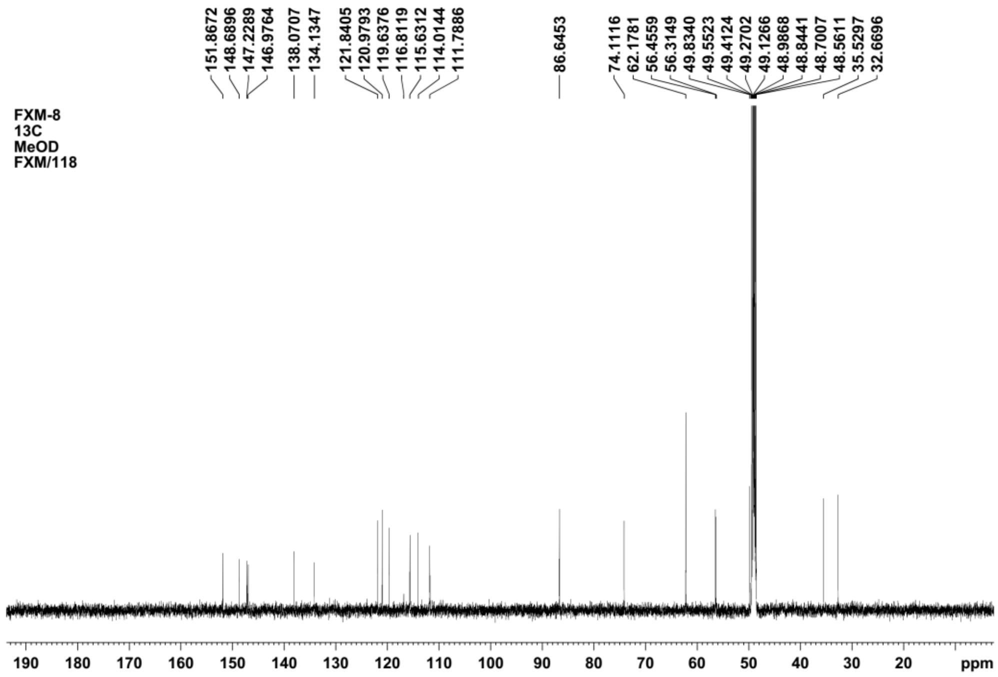

Figure S66. ^{13}C NMR spectrum of **32** (150 MHz, CD_3OD)

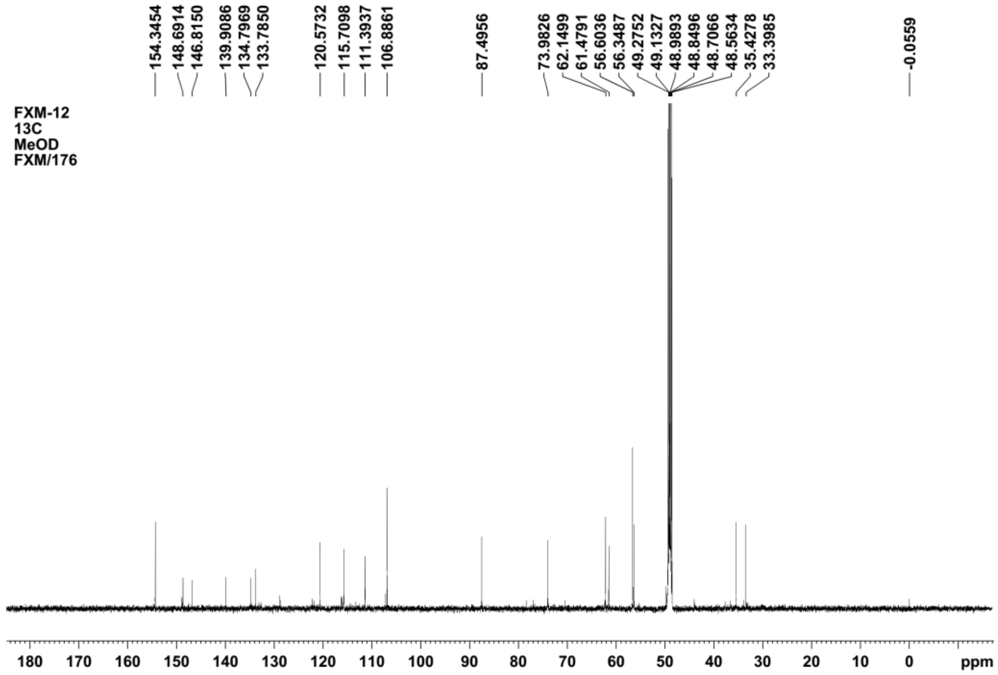

Figure S67. ^{13}C NMR spectrum of **33** (150 MHz, CD_3OD)

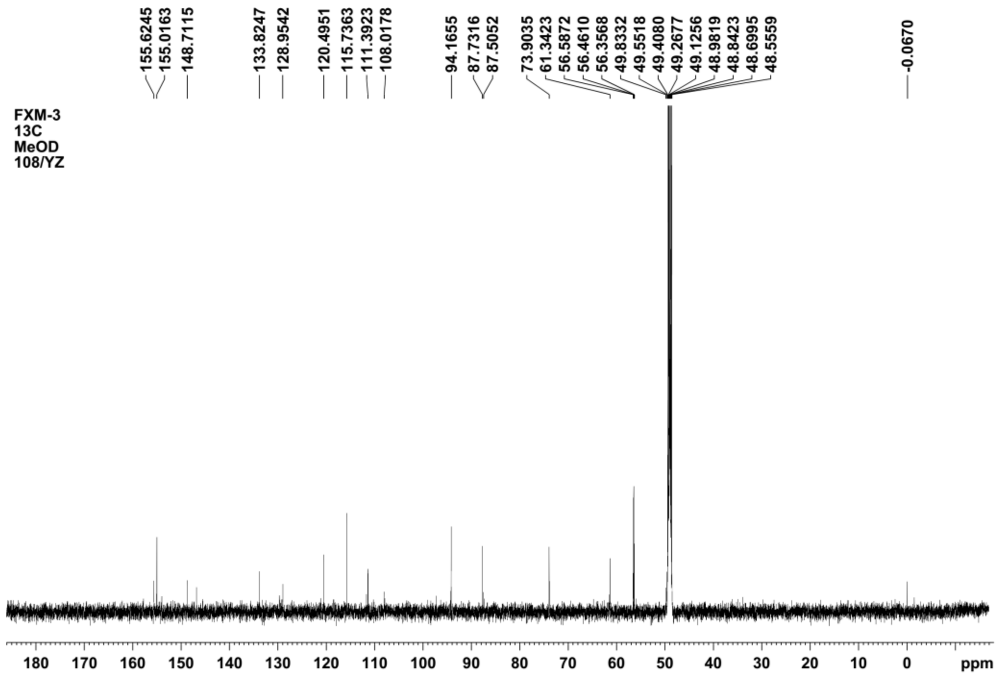

Figure S68. ^{13}C NMR spectrum of **34** (150 MHz, CD_3OD)

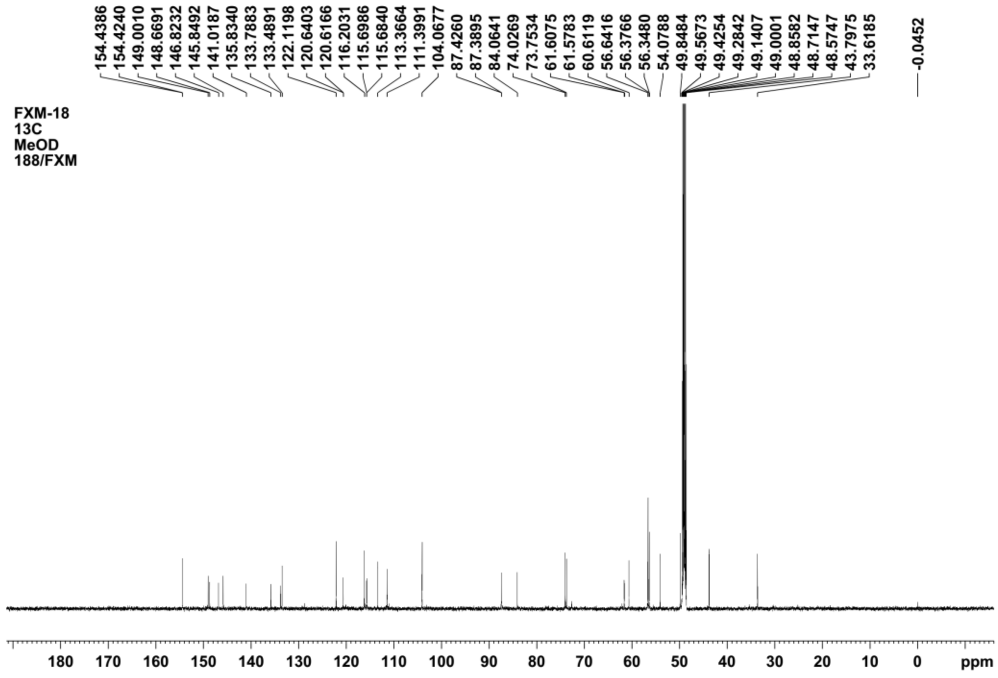

Figure S69. ^{13}C NMR spectrum of **35** (150 MHz, CD_3OD)

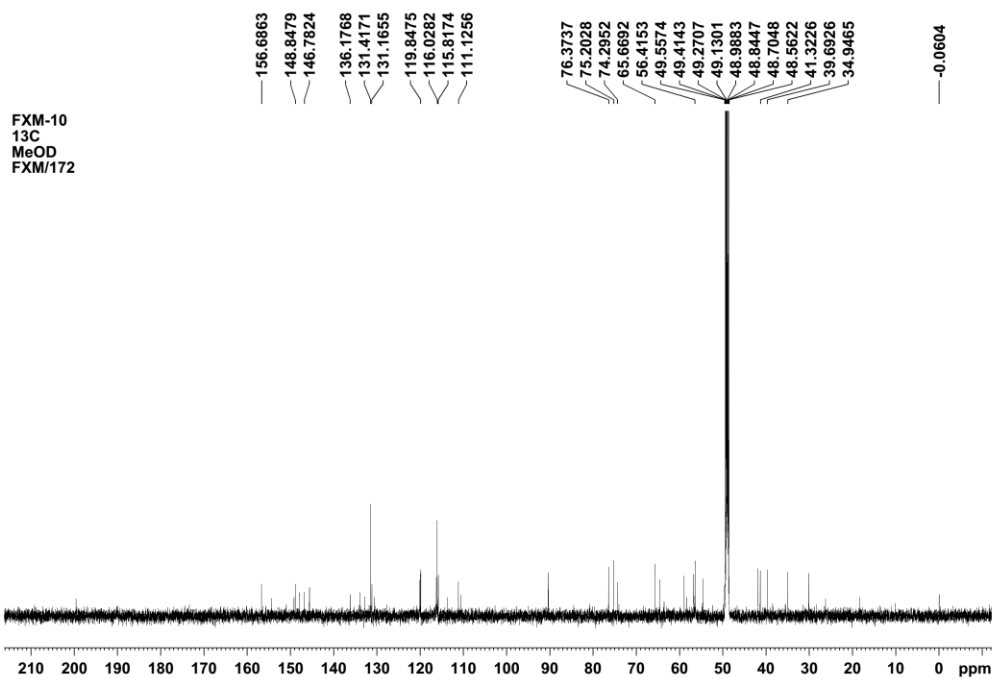

Figure S70. ^{13}C NMR spectrum of **36** (150 MHz, CD_3OD)

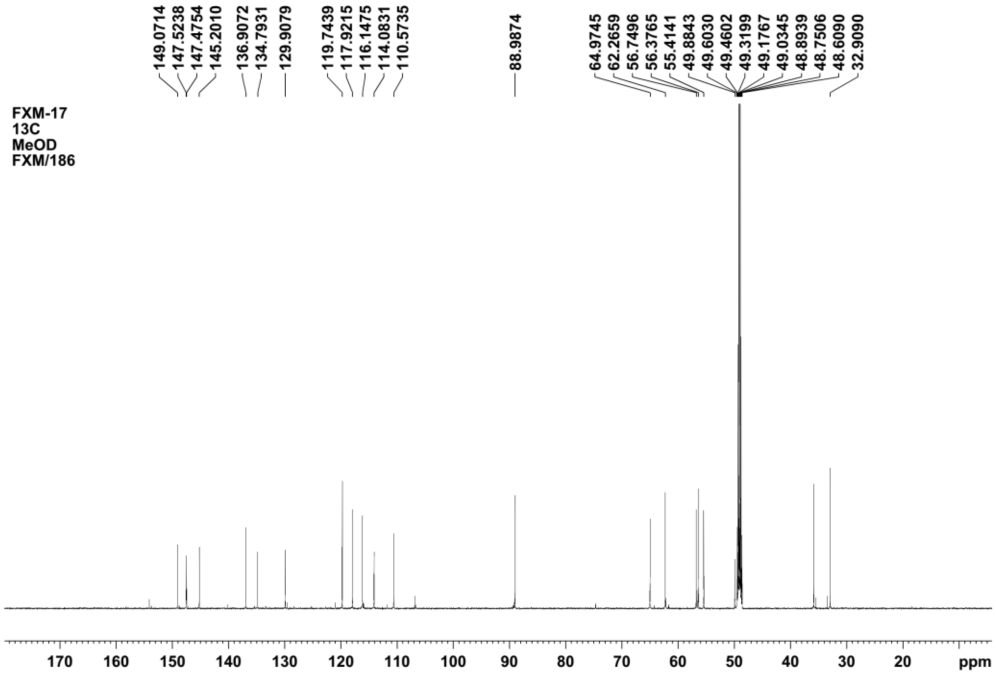

Figure S71. ^{13}C NMR spectrum of **37** (150 MHz, CD_3OD)
